# l-Arginine, as an essential amino acid, is a potential substitute for treating COPD via regulation of ROS/NLRP3/NF-κB signaling pathway

**DOI:** 10.1186/s13578-023-00994-9

**Published:** 2023-08-18

**Authors:** Chunhua Ma, Kexi Liao, Jing Wang, Tao Li, Liangming Liu

**Affiliations:** 1 State Key Laboratory of Trauma, Burns and Combined Injury, Shock and Tranfusion Research, Department of Army Medical Center, Army Medical University, Chongqing, 400042 People’s Republic of China; 2 The Affiliated Nanjing Hospital of Nanjing University of Chinese Medicine, Nanjing, 210001 China; 3 Institute of Hepatobiliary Surgery, First Affiliated Hospital, Army Medical University, Shapingba District, Gaotanyan Road 30, Chongqing, 400038 China; 4 School of Biology and Food Engineering, Institute of Pharmaceutical Biotechnology, Suzhou University, Anhui, China

**Keywords:** Metabolic markers, COPD, l-Arginine

## Abstract

**Backgrounds:**

Chronic obstructive pulmonary disease (COPD) is a frequent and common disease in clinical respiratory medicine and its mechanism is unclear. The purpose of this study was to find the new biomarkers of COPD and elucidate its role in the pathogenesis of COPD. Analysis of metabolites in plasma of COPD patients were performed by ultra-high performance liquid chromatography (UPLC) and quadrupole time-of-flight mass spectrometry (TOF–MS). The differential metabolites were analyzed and identified by multivariate analysis between COPD patients and healthy people. The role and mechanisms of the differential biomarkers in COPD were verified with COPD rats, arginosuccinate synthetase 1 (*ASS-l*) KO mice and bronchial epithelial cells (BECs). Meanwhile, whether the differential biomarkers can be the potential treatment targets for COPD was also investigated. 85 differentials metabolites were identified between COPD patients and healthy people by metabonomic.

**Results:**

l-Arginine (LA) was the most obvious differential metabolite among the 85 metabolites. Compare with healthy people, the level of LA was markedly decreased in serum of COPD patients. It was found that LA had protective effects on COPD with in vivo and in vitro experiments. Silencing *Ass-1*, which regulates LA metabolism, and α-methy-dl-aspartic (NHLA), an *Ass-1* inhibitor, canceled the protective effect of LA on COPD. The mechanism of LA in COPD was related to the inhibition of ROS/NLRP3/NF-κB signaling pathway. It was also found that exogenous LA significantly improved COPD via regulation of ROS/NLRP3/NF-κB signaling pathway. l-Arginine (LA) as a key metabolic marker is identified in COPD patients and has a protective effect on COPD via regulation of ROS/NLRP3/NF-κB signaling pathway.

**Conclusion:**

LA may be a novel target for the treatment of COPD and also a potential substitute for treating COPD.

**Supplementary Information:**

The online version contains supplementary material available at 10.1186/s13578-023-00994-9.

## Introduction

Chronic obstructive pulmonary disease (COPD) is a chronic inflammatory lung disease characterized by persistent and progressive airflow restriction [1]. Long-term exposure of respiratory tract to harmful particles such as cigarette smoke will cause repeated inflammation damage and tissue repair, which will lead to airway stenosis and continuous airflow restriction [2, 3]. There are more than 600 million COPD patients in the world at present and with the aggravation of environmental pollution, the morbidity will continuously increase, and it was once predicted that COPD would become the third leading cause of death in the world by 2020 [4]. At present, the recommended drugs for COPD treatment include bronchodilators, glucocorticoids, β-adrenergic receptor agonists, anticholinergic drugs, aminophylline and so on. But these treatments are not satisfactory. The main reason is that the pathogenesis of COPD is unclear. Therefore, it is urgent to elucidate the new mechanism of COPD and so as to find the new and more effective treatment approach to treat COPD.

The development of metabonomics, genomics and proteomics has produced effective new methods for diseases [5]. Metabonomics mainly detects small molecular compounds, including amino acids, lipids, fatty acids and carbohydrates, which are biochemical pathways related to cell physiology, structure and signal transduction [6–10]. Metabonomics has been widely used in the prevention, treatment, diagnosis and monitoring of many clinical diseases, such as Alzheimer's disease, coronary heart disease and other diseases [11, 12]. For example, Luo et al. used metabonomics to find the early biomarkers of liver cancer [13]. Li et al. used metabonomics to find the early biomarkers of colorectal cancer [14]. Because of the advantages of metabonomics in finding biomarkers of many diseases, metabonomics technology has been gradually applied to respiratory system diseases, including COPD. Recently, there were some reports about metabonomics in COPD such as the study of the severity of COPD through multi-omics [15], untargeted metabolomics of human plasma revealing lipid markers in COPD [16], predictive diagnosis of COPD using serum metabolic biomarkers and least squares support vector machines [17], direct relationship between plasma metabolomics and clinical predictors of survival difference in patients with COPD [18]. The above reports focus on the correlation between the differences in the metabonomics of COPD and the diagnosis and prediction. However, these reports were limited, they just used it to search for the changes or function of metabolites, did not investigate their in-depth mechanism. Therefore, the aims of the present study included: (1) identified the differential metabolites in COPD patients and healthy people by metabonomics analysis and searched for the functional biomarkers. (2) verified the biological function and mechanism of the biomarkers in COPD in vivo and in vitro with COPD rats, *Ass-1* KO COPD mice and bronchial epithelial cells (BEC). (3) proved whether the biomarkers can be the potential treatment targets of COPD.

## Materials and methods

### Ethical approval of the study protocol

All patient experiments were performed under the guidance of the Helsinki Declaration and approved by the Research Council of Affiliated Nanjing Hospital of Nanjing University of Chinese Medicine (KY2017019). All animal experiments were approved by the Research Council and Animal Care and Use Committee of Affiliated Nanjing Hospital of Nanjing University of Chinese Medicine and Army Medical Center, Army Medical University.

### Reagents and kits

Cigarettes were purchased from China Tobacco Guangdong industrial Co., Ltd. (Guangdong, China). Interleukin (IL)-6, IL-1β and tumor necrosis factor (TNF)-α enzyme linked immunosorbent assay (ELISA) kits were obtained from Elabscience (Wuhan, China). NLRP3, ASC, IL-1β, Caspase-1, p-NF-κB, NF-κB, p-IκBa, IκBa and GAPDH antibodies were purchased from Cell Signaling Technology. Methanol, formic acid and acetonitrile were purchased from CNW Company. L-2-chlorophenylalanine was purchased from Shanghai HengChuang Biotechnology Co., Ltd., All chemicals and solvents are analytically pure or chromatographic grade.

### COPD patients and animals

#### COPD patients

221 patients with stable COPD admitted to the Affiliated Nanjing Hospital of Nanjing University of Chinese Medicine from January 2017 to June 2019 were enrolled as the research objects, all participants provided written informed consent, and ethics approval was obtained from Affiliated Nanjing Hospital of Nanjing University of Chinese Medicine, including 150 males and 71 females. The average age was (65.5 ± 12.3) years, ranging from 62 to 75 years old. The course of disease ranged from 7 to 12 years, with an average course of (10.3 ± 3.5) years.60 healthy people were selected as control group. The average age was (64.2 ± 11.9) years, ranging from 61 to 73 years old.

#### Inclusion criteria of COPD patients

Participants of COPD patients were included according to the standards of the Global Initiative for Chronic Obstructive Lung Disease. COPD was defined by the GOLD standard of post-bronchodilator FEV1 < 80% and FEV1/FVC < 0.7, and by physician diagnosis; all were > 40 years old and had a previous history of smoking. Healthy controls were adults and > 40 years old with no history of cardiac or respiratory disease, and with normal lung function measured by spirometry (FEV1/FVC ratio > 0.7 and FEV1 > 80% predicted). Characteristics of COPD can be seen in Table 1.

**Table 1:** Basic characteristics of COPD patients

Patient characteristics	COPD
Number of patients (*n*)	221
Mean Age (years)	65.5 ± 12.3
Gender (*n*)	
(i) Women	71
(ii) Men	150
Mean body mass index (BMI) (minimum–maximum)	28.4 (17.1–56.8)
(i) Normal weight (18.5–24.9 kg/m^2^)	21.5 (18.3–24.5)
(i–ii) Normal weight (< 20 kg/m^2^)	18.4 (15 patients)
(ii) Cachexia (< 18.5 kg/m^2^)	17.1 (3 patient)
(iii) Obese (≥ 25.0 kg/m^2^)	30.3 (25.2–56.3)
GOLD stages (*n*)	
Stage I	42
Stage II	125
Stage III	34
Stage IV	20
Smoking history (*n*)	
Current smoker	142
Never smoker	77
Ex-smoker	2

#### Exclusion criteria of COPD patients

Participants were excluded from the study due to any antibiotic or oral corticosteroid use in the past 4 weeks, a current smoker, had comorbid lung disease (e.g., asthma, lung cancer, interstitial lung disease and bronchiectasis) that interferes with the study outcomes, had other co-morbidities with established altered microbiome (including IBD, irritable bowel syndrome), or extreme dietary habits that may significantly impact gut microbiome composition.

### Animals

The Sprague–Dawley (SD) rats were obtained from Army Medical Center, Army Medical University. WT mice (C57BL/6J, stock No: 000663) and *Ass-1* KO mice (C57BL/6J, *Ass-1* ^−/−^/J stock No: 020289) were obtained from the Cyagen Biosciences Inc. and kept in the Animal Center of Army Medical Center, Army Medical University.

### COPD patient confirmation and acquisition of samples

#### CT analysis

COPD patients and healthy people were scanned with Siemens Somatom E-motion 6 multi-slice spiral CT machine in supine position from the tip of lung to the joint lower edge of lung bottom. The scanning layer was set to 1.25 mm, the voltage was set 120 kV, and the current was set to 240–300 mA. The special workstation was used to process the obtained images. Total emphysema volume (the total emphysema volume, TEV) and the total lung volume (TLV) were recorded. The emphysema index (EI) was calculated according to the emphysema parameters, the formula was EI = TEV/TLV.

#### Pulmonary function

The changes of lung function indexes in two groups were recorded: maximum expiratory flow (PEF), forced vital capacity (FVC) and the percentage of forced expiratory volume in the first second (FEV1%).

#### Serum collection and plasma collection

For the serum, 3 mL of peripheral venous blood was taken in a non-anticoagulant tube under empty stomach condition from COPD patients and healthy people. The obtained blood was centrifuged at 168 g/min, 4 °C for 10 min, and the serum was collected for IL-1β, IL-6 and TNF-α detection. For the plasma, 3 mL of peripheral venous blood was collected in an anticoagulant tube under empty stomach condition from COPD patients and healthy people. The obtained blood was centrifuged at 168 g/min, 4 °C for 10 min, and the plasma was collected for metabonomics analysis.

### COPD model establishment in rats and *Ass-1* KO mice

#### COPD animal model preparation

The mice model was established as previously described [19], Except for control group, briefly, ten mice in COPD group were exposed to 10 cigarettes (China Tobacco Guangdong industrial Co., Ltd. (Guangdong, China), 12 mg of tar and 0.9 mg of nicotine) two times per day with 30 min smoke-free intervals in a closed 0.75-m^3^ room, 5 days per week for up to 90 days. Mice tolerated CS (cigarette smoking) exposure without evidence of toxicity (carboxyhemoglobin levels∼10% and no weight loss). An optimal smoke: air ratio of 1:6 was obtained. Ten mice in control group were exposed to air. The diet, drinking water, environment and other factors of control mice and CS mice were the same, so as to exclude the influence of nutritional factors and environmental factors on metabonomic detection. Mice were divided into the following groups: (1) WT mice, (2) KO mice, (3) WT mice + CS, (4) KO mice + CS. On the 91st day, mice were tested for airway reaction, on the 92nd day, blood and tissue samples were collected.

The CS-induced COPD rat model was established as previous report [20]. Rats in the CS inhaled CS for 90 days using the BUXCO system (Data Sciences International, New Brighton, NM, USA). The smoke was produced by Derby cigarettes (Wuhu Cigarettes, China). Each cigarette contains 10 mg tar, 0.9 mg cotinine, and 12 mg carbon monoxide. Rats were treated with inhalation of the equivalent of 20 cigarettes for 2 h, and then allowed to rest for 4 h, which was repeated again on the same day. Rats inhaled CS for six days/week. Sham rats breathed air using the BUXCO animal CS-exposure system for 90 days, then breathed air in a 20-L plastic chamber for seven days. Rats tolerated CS (cigarette smoking) exposure without evidence of toxicity (carboxyhemoglobin levels ∼10% and no weight loss). The diet, drinking water, environment and other factors of control rats and CS rats were the same, so as to exclude the influence of nutritional factors and environmental factors on metabonomic detection. Rats were divided into the following groups: (1) control rats, (2) CS rats. On the 91st day, mice were tested for airway reaction, on the 92nd day, blood and tissue samples were collected.

#### Alveolar lavage fluid (BALF) collection from COPD rats and *Ass-1* KO mice

COPD rats and *Ass-1* KO mice were anesthetized by intraperitoneal injection of 10% chloral hydrate (35mg/kg). The limbs and head of the rats and mice were fixed. After disinfection, the chest cavity was opened to expose the trachea of the animals, and a small T-shaped incision was transversely and longitudinally cut at the lower end of the trachea. The cannula was inserted along the incision, and then ligated and fixed. 2 mL of preheated normal saline (37 °C) was slowly injected into the lungs of animals through inserted catheter, and then gently massaged the chest of animals. After 30 s, the lavage fluid was withdrawn and injected back into the lungs, and then collected the BALF. The obtained BALF was used for the detection of IL-1β, IL-6 and TNF-α.

### Serum collection and analysis

COPD rats and *Ass-1* KO mice were anesthetized by intraperitoneal injection of 10% chloral hydrate (35mg/kg). Blood was obtained via common carotid artery in COPD rats and via orbital vein in COPD *Ass-1* KO mice. The obtained blood was centrifuged at 168 g/min, 4 °C for 10 min, and the supernatant (serum) was collected for IL-1β, IL-6 and TNF-α and LA detection.

### Lung tissues collection and analysis

After COPD rats and *Ass-1* KO mice were sacrificed, the left lung (because the two lung branches were ligated when the alveolar lavage fluid (BALF) was collected, and one lung (right lung) was used for alveolar lavage, the other lung (left lung) was used for the detection of indicators to avoid the impact of BALF on the detection of indicators) was carefully removed on the crushed ice. Part of it was used for pathological examination, including HE staining and Masson staining, part of it was used for detecting the reactive oxygen species (ROS), nitric oxide (NO) and type I collagen (Collagen I).

### Cigarette smoke extract-induced injury of bronchial epithelial cells and analysis

#### Extraction and separation of bronchial epithelial cells (BECs)

According to previous report [21], health mice were anaesthetized using 2% isoflurane inhalation and killed by cervical dislocation. The bronchus was isolated from lobes of lung, minced to small pieces and digested by 0.05% pronase (Sigma, MA, USA) in DMEM/F12 media (Invitrogen, CA, USA) at 4 °C overnight. Digestion was stopped by adding FBS (Gibco, CA, USA). The identification method and process of BEC and the identification results were shown in Additional file 1: Fig. S1.

#### Preparation of cigarette smoke extract (CSE)

The smoke produced by the complete combustion of 20 commercial cigarettes were fully mixed and dissolved in 20 mL serum-free DMEM medium, and then filtered with 0.22 μm filter membrane to obtain the DMEM mother liquor containing CSE.

#### BECs culture

The isolated BECs were inoculated in serum-free complete F12 medium in 1 × 10^5^/60 mm plates, and cultured in 5% CO_2_ incubator at 37 °C. After 24 h, one culture plate was taken and the culture solution was discarded, the adherent cells were counted, and the adherent rate was calculated. The other culture plates were continued cultured to day 5 to harvest the cells. At day 3 the culture medium needed to be changed one time.

#### Establishment of CSE-induced BECs injury model

BECs were cultured in DMED medium containing 10% FBS in a cell incubator containing 5% CO_2_ at 37 °C. BECs were stimulated with CSE in a final concentration of 20% for 48 h.

#### Knockdown of *Ass-1* in BECs

BECs were transfected with *Ass-1* siRNA (Shanghai GenePharma, China) or negative control (NC) siRNA (Shanghai GenePharma, China) as the method mentioned in the instructions. The sequences of *Ass-1* siRNAs are listed in Additional file 2: Fig. S2. Lipofectamine 2000 was used for transient siRNA transfections (Invitrogen) according to the manufacturer's recommendation. A complex of 0.5 mL Opti-MEM medium and 6 μL of Lipofectamine 2000 containing 50 nM *Ass-1* siRNA or 50 nM NC were premixed per well in a 6-well plate.

#### Ass-1 inhibitor experiment

BECs were inoculated in 6-well plate at the density of 1 × 10^5^ cells/well, and cultured in constant temperature incubator at 37 °C, BECs were stimulated with CSE in a final concentration of 20% for 48 h and then incubated with *Ass-1* inhibitor NHLA with final concentration of 10, 25, 50 nM for 24 h.

### Culture solution collection and analysis

Culture solution from CSE-stimulated BECs, *Ass-1* knocked down BECs and *Ass-1* inhibitor treatment BECs was centrifuged at 168 g /min, 4 °C for 10 min and the supernatant was collected for IL-1β, IL-6 and TNF-α detection.

### BECs collection and analysis

CSE-stimulated BECs, *Ass-1* silenced BECs and arginase inhibitor treated BECs were collected. Part of the cells was used to extract protein, and the protein concentration was determined by bicinchoninic acid assay (BCA). The other part of cells was used to determine the ROS and NO.

### Protective effect of exogenous LA on COPD mice and CSE-induced BECs

#### Establishment of COPD model and LA intervention in mice

The establishment method of COPD mouse model was the same as that in item “COPD animal model preparation”. Mice were divided into the following groups: (1) control mice, (2) COPD mice, (3) COPD mice + LA (100 mg/kg). LA was dissolved with normal saline, and administered by gavage immediately. ROS, NO, cytokine, pathological changes, signal pathway proteins was measured according to “Detection methods of related variables” item.

#### Establishment of COPD model and LA intervention in BECs

The establishment method of COPD mouse model was the same as that in item “Establishment of CSE-induced BECs injury model”. The BECs were divided into the following groups:(1) control, (2) CSE, (3) CSE+ LA (1 mol/L). BECs were stimulated with CSE in a final concentration of 20% for 48 h and then incubated with LA with final concentration of 1 mmol/L for 24 h. ROS, NO, cytokine and signal pathway proteins detection was measured according to “Detection methods of related variables” item.

### Detection methods of related variables

#### Plasma metabonomic of COPD patients

Before detection, the plasma sample was thawed at 4 °C. 100 μL of the sample was taken, 300 μL of acetonitrile was added, and the mixture was centrifuged at 13,400*g* for 10 min and the supernatant was dried with nitrogen, and the residue was dissolved in 100 μL of mobile phase centrifuged at 6800×*g* for 10 min, and then filtrated with a 0.22 μm filter membrane. 60 sera from 60 healthy people were finally mixed into 8 samples for metabolomics experiment, while 221 sera from 221 COPD patients were finally mixed into 8 samples for metabolomics experiment. The metabolites were detected by the chromatographic column was Waters Acquity UPLC BEH C18 column (2.1 mm × 100 mm, 1.7 μm). The mobile phase was 0.1% formic acid (A) − 0.1% formic acid-acetonitrile (B), the column temperature was 40 °C, and the injection chamber temperature was 4 °C. The ladder degree elution was set as 0–1 min, 98% A, 2%B; 1–5 min, 98% A − 93% A, 2B%–7%B; 5–12 min, 93% A − 65% A, 7%B − 35%B; 12–22 min, 65% A − 50% A, 35%B–50%B; 22–36 min, 50% A, 50%B; the flow rate was 0.25 mL/min, the injection volume was 5 μL. The mass spectrometry adopted electrospray ion source (ESI) with positive and negative ion scanning mode. The scanning range (m/z) was 50–1000, the capillary voltage was 3.0 kV, the ion source temperature was 120 °C, the taper hole voltage was 35 V, the desolvention gas was high purity nitrogen, the desolvention temperature was 300 °C, the volume flow of desolvation gas was 600 L/min, the volume flow rate of cone hole gas was 70 L/h, the collision gas argon and collision energy were 6 V.

### Cell viability

BECs were inoculated in 96-well plate at the density of 1 × 10^4^ cells/well, and cultured in constant temperature incubator at 37 °C, BECs were stimulated with CSE in a final concentration of 20% for 48 h. After the culture medium was discarded, 150 μL dimethyl sulfoxide (DMSO) was added. The absorbance value of each well was determined with a microplate spectrophotometer at 490 nm.

### Airway reactivity detection in COPD rats and *Ass-1* KO mice

The airway reactivity of COPD rats and *Ass-1* KO mice were measured by invasive lung impedance method with Buxco lung function detector. Briefly, the animals were anesthetized by intraperitoneal injection of 4% chloral hydrate at 0.1 mL/10 g, a proper endotracheal intubation was performed and the ventilator was used. The frequency of ventilator was set to 120 times/min. The airway resistance of rats and mice was obtained by measuring the changes of airway airflow and pressure. Firstly, the basic value of animal’s airway resistance was recorded for l min. Then, the changes of airway resistance were measured after atomization of l0 μL acetylcholine (Ach) for twice application, each time lasted for 1 min, the recording time lased for 3 min. The concentration of acetylcholine was at 0, 6.25, 12.5 and 25 mg/mL from low to high. The changes of the resistance of lung (RL) and lung dynamic compliance (Cdyn) of animals was calculated.

### IL-1β, IL-6 and TNF-α detection in serum, BALF of COPD patients, rats and KO mice and cell supernatant of BECs

Enzyme-linked immunosorbent assay (ELISA) was used to detect the contents of IL-1β, IL-6 and TNF-α in serum, BALF of COPD patients, rats, KO mice and cell supernatant of BECs. All the operation steps were strictly performed in accordance with the instructions of ELISA kits.

### ROS detection lung tissues of COPD rats, *Ass-1* KO mice and BECs

20 mg of lung tissue of COPD rats and COPD KO mice were homogenized with 9 times of normal saline, and then centrifuged at 4 °C for 13400 g for 10 min to prepare single cell suspension for later use. Single cell suspension of lung tissue and bronchial epithelial cells were inoculated into 24-well plates. When the cell density reached about 80%, 10 μmol/L DCFH-DA was added to each well, and cultured at 37 °C for 30 min. Five fields were randomly selected under fluorescence microscopy, and the green fluorescence intensity was analyzed. The mean fluorescence intensity (MFI) could indirectly reflect the ROS level.

### Determination of NO and Collagen I in lung tissue of COPD rats, *Ass-1* KO mice

100 mg lung tissue of COPD rats and *Ass-1* KO mice was homogenized in PBS buffer solution (pH = 7.4) at a ratio of 1: 5 under ice bath. The homogenate was centrifuged at 4 °C for 13,400*g* for 10 min. The supernatant was collected to detect the contents of NO and collagen I according to instructions of corresponding reagent kits.

### HE staining of lung tissue of COPD rats and *Ass-1* KO mice

After dehydration and paraffin embedding, the fixed lung tissue of COPD rats and *Ass-1*KO mice was cut into paraffin section with a thickness of 5 μm and stained with hematoxylin and eosin (HE) and then observed under a light microscopy (Nikon, Tokyo, Japan) at 200 × magnification.

### Masson staining of lung tissue of COPD rats and *Ass-1* KO mice

Lung tissue was fixed by 4% paraformaldehyde and then subjected to routine operations such as dehydration, embedding and slicing. After obtaining slices, the slices were subjected to Masson staining according to the instructions of the kit. Finally, the slices were sealed with neutral resin. After the slices were dried, they were observed with microscope.

### Immunohistochemistry staining

The expressions of p-NF-κBp65 and NLRP3 in lung tissues of COPD rats and *Ass-1* KO mice were evaluated by immunohistochemistry. Briefly, the lung tissue was fixed with 4% paraformaldehyde (PFA), embedded in paraffin and then sectioned. The paraffin sections were dewaxed in xylene and dehydrated ethanol, microwaved (15 min and 800W) in sodium citrate buffer and washed with PBS. Endogenous peroxidase activity was blocked by 3% hydrogen peroxide for 20 min. Each sample was blocked with 5% goat serum for 20 min and then treated overnight with p-NF-κB (1:200), NLRP3 (1:200) antibodies at 4 °C. On the next day, each sample was washed three times with PBS before treated with goat anti-rabbit IgG secondary antibody for 20 min. They were then washed three times with PBS afterwards. Next, samples were stained with 3–3’diaminobenzidine (DAB) and later stained with hematoxylin. After dehydrated and dried, the sections were mounted with neutral gum and observed with microscope.

### Immunofluorescence staining

The levels of NLRP3 and p-NF-κB in BECs were evaluated by immunofluorescence. Briefly, cultured BECs were washed twice with PBS, fixed with 4% paraformaldehyde (PFA) for 30 min, and then permeabilized with 0.5% Triton X-100 in PBS for 5 min, blocked with 5% BSA for 1 h. The cells were incubated with the primary antibodies NLRP3 (1:500) and p-NF-κB (1:500) overnight at 4 °C, washed three times with PBS and incubated with goat anti-rabbit IgG secondary antibody, Alexa Fluor® 488 conjugate (1:500) for 1 h. After three times washing with PBS and the DAPI was done at room temperature for 5 min. Fluorescence images were taken with fluorescence microscopy.

### Western blotting

Cell lysate (containing 1% PMSF and 1% phosphorylated protease inhibitor) was added into the lung tissue of COPD rats and *Ass-1* KO mice and BECs samples. The lysate was centrifuged at 12 000 r/min for 15 min, and the supernatant was collected to extract the total cell protein. After detecting the protein concentration by Bicinchoninic Acid Assay (BCA) method, the protein sample was mixed with 5 × loading buffer at a ratio of 4:1, and then boiled at 100 °C for 5 min. Protein samples were separated by SDS–polyacrylamide gel electrophoresis, and then transferred to polyvinylidene fluoride membrane at a constant current of 230 mA. The membrane was placed in 5% skim milk powder, sealed at room temperature for 90 min, and diluted primary antibody was added and incubated at 4 °C overnight. The primary antibody concentration was NLRP3 (1:1000), ASC (1:1000), Caspase-1 (1:1000), p-NF-κBp65 (1:1000), NF-κB (1:1000), p-IκBa (1:1000), IκBa (1:1000) and GAPDH (1:1000). After washing the membrane with 1 × TBST, the second anti-goat anti-rabbit IgG (diluted 1: 10,000) was added and incubated for 1 h at room temperature. After washing the film again, enhanced chemiluminescent (ECL) solution was added and the gray value of the strip was analyzed with Image Lab software.

### Molecular docking

PDB database (http://www.rcsb.org/) was used to search the protein structure of the target, and then PyMOL software was used to remove water and phosphate from the target. at the same time, AutoDuck Tools software was used to hydrogenate and charge the receptor, and the output was in recognizable Pdbqt format. AutoDuck Vina software was used to connect the NLRP3 with LA.

### Statistical analysis

SPSS 23.0 statistical software was used to analyze the data, and the measurement data was expressed by mean ± SD. One-way variance analysis was used to compare the mean of multiple samples, and *P* < 0.05 indicated that the difference was statistically significant.

## Results

### Differential metabolites identification

#### Confirmation of COPD patients

**Fig. 1:**
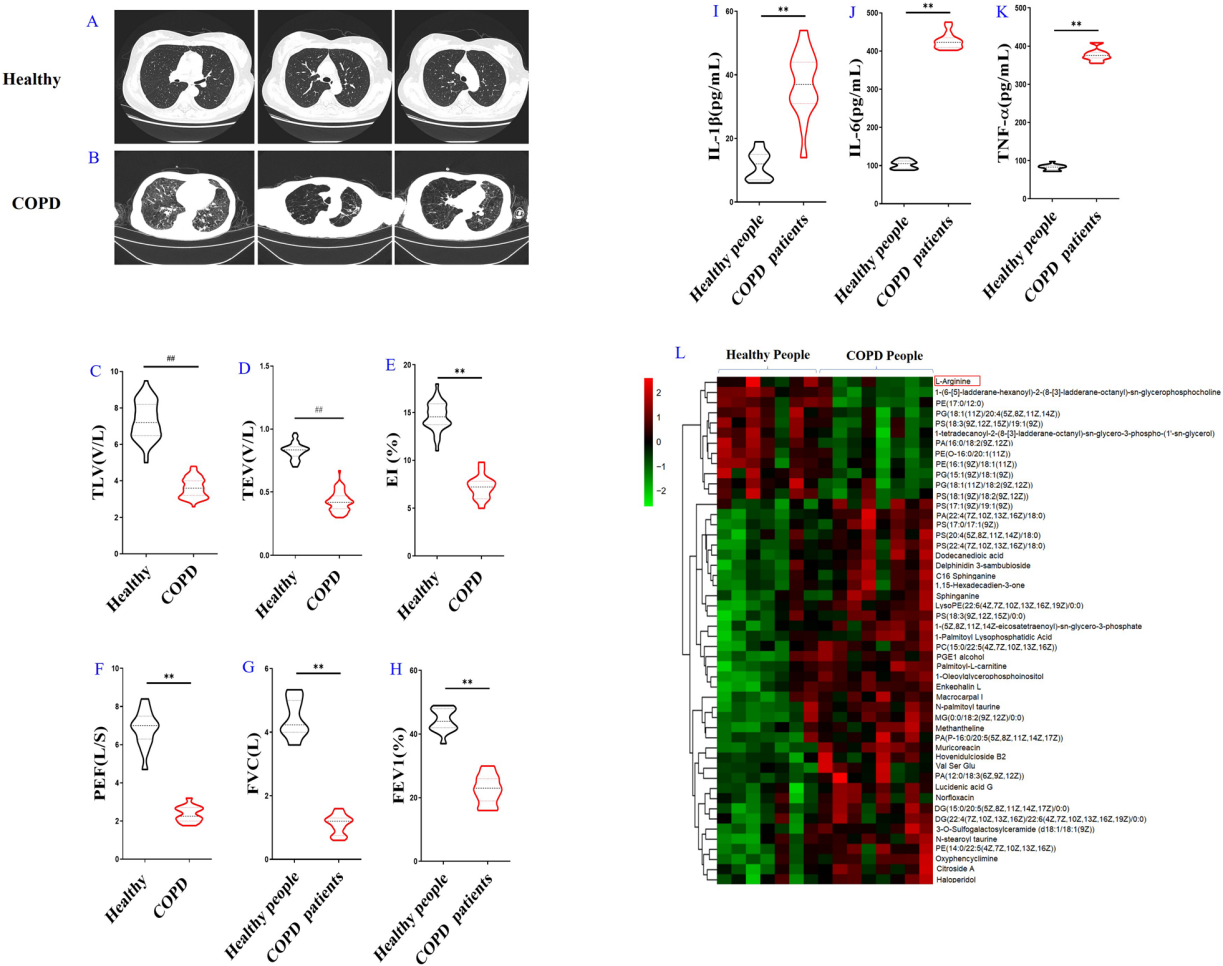
Confirmation and metabonomics of COPD patients. Typical Computed Tomography (CT) of COPD patients (**A**) and healthy people (**B**). Parameters of CT analysis of COPD patients: Total Emphysema Volume (TEV) (**C**), Total Lung Volume (TLV) (**D**) and Emphysemaindex (EI) (**E**). Pulmonary function parameters of COPD patients: Maximum Expiratory Flow (PEF) (**F**), Forced Vital Capacity (FVC) (**G**), the percentage of forced expiratory volume in the first second (FEV1%) (**H**). Serum cytokine levels in COPD patients by Enzyme linked immunosorbent assay (ELISA): interleukin-1β (IL-1β) (**I**), interleukin-6 (IL-6) (**J**), tumor necrosis factor-α (TNF-α) (**K**). The Top 50 differential metabolites were identified and the results are shown in the form of heat map: the red part represents metabolites with relatively high content, and the green part represents metabolites with relatively low content (**L**). (n = 211 for COPD patients and n = 60 for healthy people). All data were presented as mean ± SD. Compared with healthy people: ^##^P < 0.01

CT, as one of the important means of clinical diagnosis of COPD, is helpful to make more reasonable treatment plans [22]. Therefore, in this study, COPD patients were all examined by CT. The image features of healthy people were bilateral thoracic symmetry distribution, normal lung permeability, clear lung texture, no thickening and disorder. While, COPD patients showed thickened lung texture, disorder lung texture, twisted, thickened bronchial wall, increased lung volume, and increased lung brightness (Fig. 1A–E and Additional file 1: Fig. S1). These results further confirmed the diagnosis of COPD for the enrolled patients.

The detection of pulmonary function is very important for the diagnosis of COPD. The results showed that the FVC, FEV1% and PEF in COPD patients were all significantly decreased as compared with the healthy people (Fig. 1F–H).

Inflammatory reaction is an important feature of COPD. In this study, serum cytokines in COPD patients were examined. Compared with healthy people, the contents of IL-1β, IL-6 and TNF-α in serum of COPD patients were significantly increased (Fig. 1I–K).

### Plasma metabonomic of COPD patients

As shown in Additional file 1: Fig. S1, the plasma metabolic fingerprints of COPD patients were obtained. The results showed that these metabolites were mainly concentrated in fatty acids and amino acids, the molecular weight of these metabolites were most less than 1000 kD. The retention time of these metabolites was mainly concentrated in 0–10 min. The metabolites were analyzed by principal component analysis (PCA), partial least squares-discriminant analysis (PLS-DA), orthogonal partial least squares-discriminant analysis (OPLS-DA) and response ranking test of OPLS-DA model. As shown in Additional file 3: Fig. S3. 85 differential metabolites were identified in this study (Additional file 5: Table S1). Also, the 85 differential metabolites were mainly fatty acids and amino acids. In order to accurately know the content change of the 85 metabolites in COPD patients, we quantitatively compared the 85 metabolites. As shown in (Additional file 6: Table S2), as compared with the healthy people, two-thirds of the 85 differential metabolites were increased, and one-third of the 85 differential metabolites were decreased, among which the content of L-arginine (LA) was the most decreased one.

In order to further clarify the greater differential metabolites among the 85 differential metabolites, TOP50 analysis method was used to analyze the 85 metabolites to obtain the TOP 50 metabolites according to variable important in projection (VIP) value, which was calculated from their contents. As shown in Fig. 1P, for the Top 50 differential metabolites, the hot spot analysis was used according to VIP value. The results showed, among them, LA had the largest difference. Results showed that through metabolomics analysis and bioinformatics analysis, we found LA was the largest changeable metabolite. In addition, serum LA in patients with COPD decreased significantly compared with healthy people (Additional file 4: Fig. S4).

## Verification of the role of LA in COPD

### In COPD rats

#### RL was increased and Cdyn was decreased in COPD rats

**Fig. 2:**
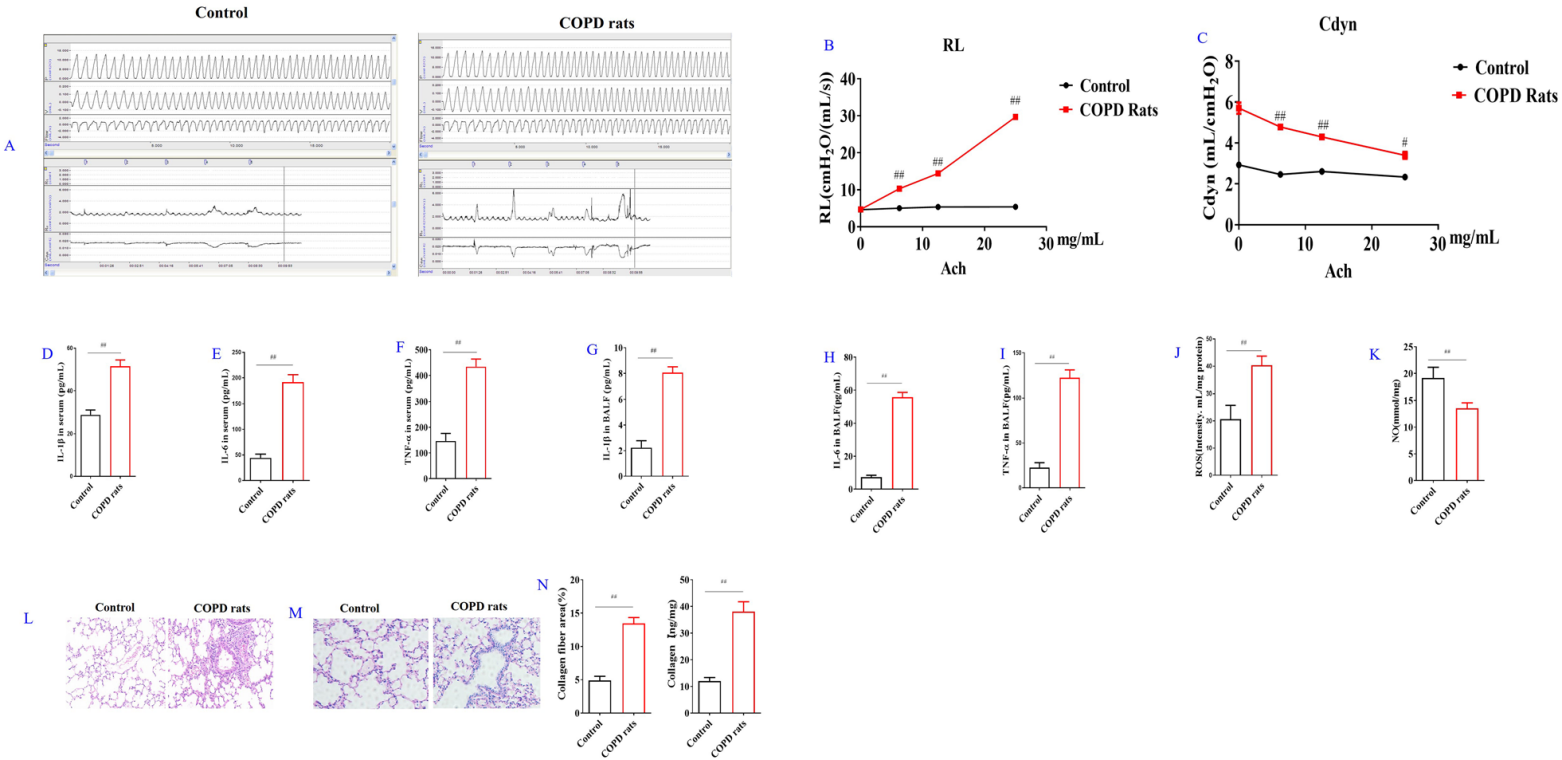
Effects of l-arginine (LA) on COPD rats. Airway reaction (**A**). The percentage changes of the resistance of lung (RL) (**B**) and lung dynamic compliance (Cdyn) (**C**) in control rats and COPD rats. Serum cytokines: The contents of interleukin-1β (IL-1β) (**D**), interleukin-6 (IL-6) (**E**), tumor necrosis factor-α (TNF-α) (**F**). BALF cytokines: the contents of Interleukin-1β (IL-1β) (**G**), interleukin-6 (IL-6) (**H**), tumor necrosis factor-α (TNF-α) (**I**). Reactive oxygen species (ROS) (**J**) levels and nitric oxide (NO) (**K**) content of lung in COPD rats. Pathological changes (HE staining) and Masson staining of lung in COPD rats: HE staining of lung in COPD rats (× 200) (**L**), Masson staining of lung in COPD rats (× 200) (**M**), Collagen quantification of Masson staining and collagen I contents of lung in COPD rats (**N**). (n = 10). All data were presented as mean ± SD. Compared with control rats: ^##^P < 0.01

Airway stenosis is one of the important pathological features of COPD [23]. Therefore, the airway reactivity of COPD rats was detected (Fig. 2A). As compared with control rats, the level of RL in COPD rats was significantly increased after Ach stimulation at 6.25, 12.5 and 25 mg/mL (Fig. 2B). While the level of Cdyn in COPD rats was significantly decreased as compared with the control rats after Ach stimulation at 6.25, 12.5 and 25 mg/mL (Fig. 2C).

### IL-1β, IL-6 and TNF-α were increased in serum and BALF in COPD rats

Inflammation is the critical mechanism for COPD development, inflammatory mediators and destructive enzymes released by inflammatory cells play important role in the progressive destruction of the lung in COPD [24]. The contents of IL-1β, IL-6 and TNF-α in serum and BALF in COPD rats were detected in the present study. The results showed that the concentration of IL-1β, IL-6 and TNF-α in serum and BALF of COPD rats was significantly increased as compared with the control group (Fig. 2D–I).

### ROS was increased and NO was decreased in lung tissues of COPD rats

It was reported that LA has inhibitory effect on ROS and may reduce its production [25]. Meanwhile, LA, as an essential amino acid can be catalyzed as several bioactive molecules such as nitric oxide (NO), proline, creatine, and polyamines by nitric oxide synthase (NOS), arginine decarboxylase and arginase (ARG) [26]. ROS and NO contents of lung tissues in COPD rats were detected. The results showed that as compared with control rats, the level of ROS in lung tissue in COPD rats was significantly increased, the NO contents were significantly decreased (Fig. 2J, K).

### CS stimulated lung histopathological changes in COPD rats

Pathological examination of COPD is an important means to evaluate COPD diseases. Therefore, HE and Masson staining were used to evaluate the lung pathology of COPD rats. As for HE staining, the alveoli of control rats were intact, while the alveolar structure of COPD rats was incomplete. In addition, the lung bronchus of control rats was normal in shape, only a small number of inflammatory cells infiltrated around it. While in COPD rats, the lung bronchus had some lumen stenosis, and a large number of inflammatory cells infiltrated around it (Fig. 2L).

Six regions of Masson staining images were randomly selected under 100 times microscope, and the semi-quantitative analysis of the deposition ratio of collagen fibers was carried out by Image J software. The results showed that a small amount of collagen fibers was deposited around the lung bronchus in the control rats, while in COPD rats, there were more collagen fibers deposited around the lung bronchus and collagen I contents was increased (Fig. 2M, N).

### In *Ass-1* KO mice

#### *Ass-1* KO increased RL and decreased Cdyn

**Fig. 3:**
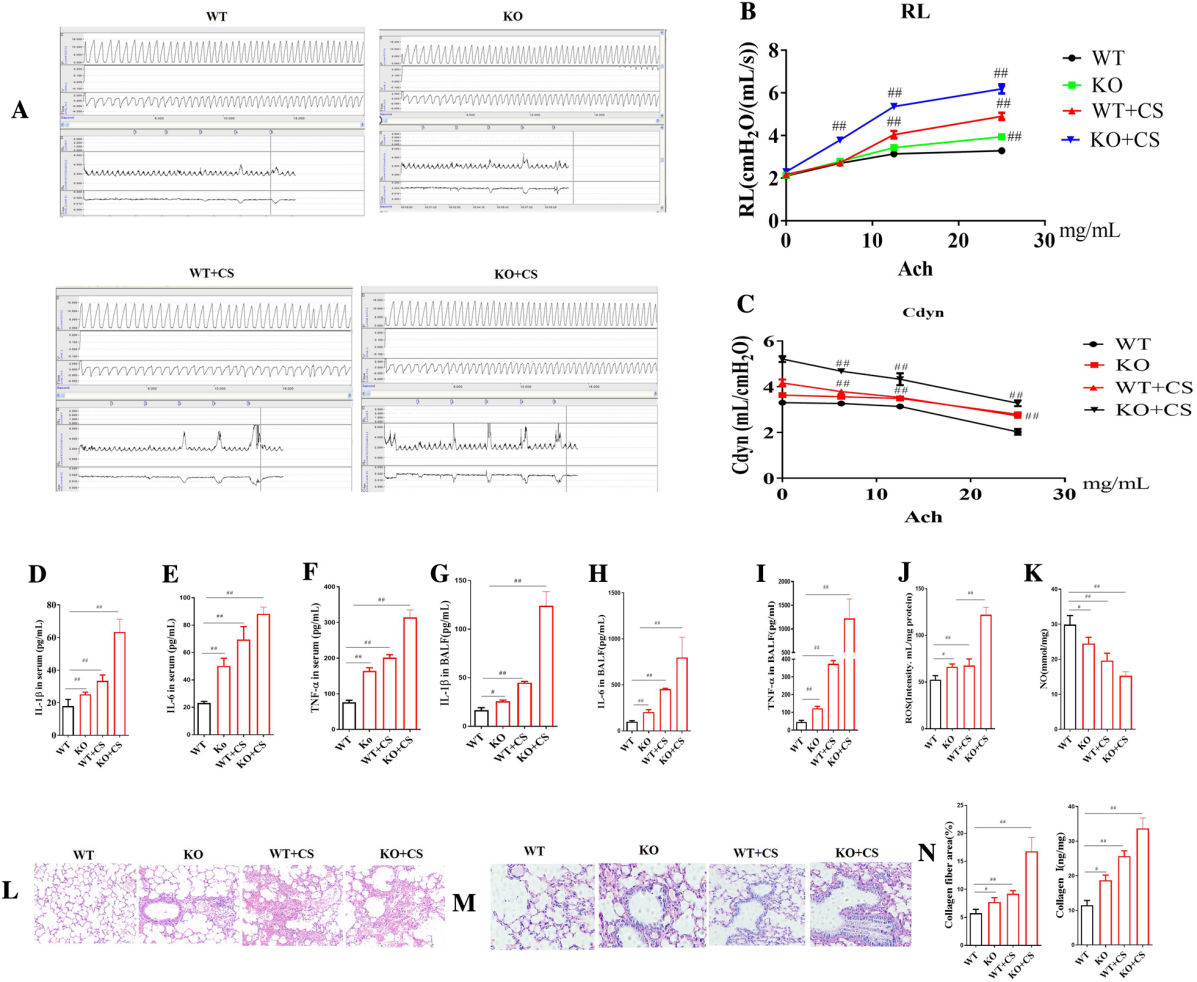
Effects of l-arginine (LA) on KO COPD mice. Airway reaction (**A**). The percentage changes of the resistance of lung (RL) (**B**) and lung dynamic compliance (Cdyn) (**C**) in WT and KO COPD mice. Serum cytokines: The contents of interleukin-1β (IL-1β) (**D**), interleukin-6 (IL-6) (**E**), tumor necrosis factor-α (TNF-α) (**F**). BALF cytokines: the contents of Interleukin-1β (IL-1β) (**G**), interleukin-6 (IL-6) (**H**), tumor necrosis factor-α (TNF-α) (**I**). Reactive oxygen species (ROS) (**J**) and nitric oxide (NO) (**K**) contents in lung tissues. Pathological changes (HE staining) and Masson staining of lung in COPD rats: HE staining of lung in COPD rats (× 200) (**L**), Masson staining of lung in COPD rats (× 200) (**M**), Collagen quantification of Masson staining and collagen I contents of lung in COPD KO mice (**N**). (n = 10). All data were presented as mean ± SD. Compared with WT mice: ^##^P < 0.01

In order to further verify the role of LA in COPD, WT and *Ass-1* KO mice were used in the present study. We detected the LA change in the serum of *Ass-1* KO mice and the changes of airway reactivity. The results showed that the concentration of LA in *Ass-1* KO mice serum was significantly decreased as compared to WT mice (Additional file 4: Fig. S4). For the RL, as compared with WT mice, RL in COPD mice was significantly increased after Ach stimulation at 12.5 and 25 mg/mL. As compared with COPD mice, RL in *Ass-1* KO COPD mice was further increased after Ach stimulation at 6.25, 12.5 and 25 mg/mL. For the Cdyn, as compared with WT mice, Cdyn in COPD mice was significantly decreased after Ach stimulation at 6.25, 12.5 and 25 mg/mL. As compared with COPD mice, Cdyn in *Ass-1* KO COPD mice was further decreased at Ach 6.25, 12.5 and 25 mg/mL (Fig. 3A–C).

#### *Ass-1* KO increased IL-1β, IL-6 and TNF-α in serum and BALF

As the same as in COPD rats, the serum IL-1β, IL-6 and TNF-α in *Ass-1* COPD mice were significantly increased as compare with WT control mice. As compared with WT COPD mice, the serum contents of IL-1β, IL-6 in *Ass-1* KO COPD mice was further increased. The change trend of IL-1β, IL-6 and TNF-α in BALF in WT COPD and *Ass-1* KO COPD mice were the same as in serum in WT COPD and KO COPD mice (Fig. 3D-I).

#### *Ass-1* KO increased ROS and decreased NO in lung tissue

In order to verify the effect of LA on ROS and NO, the *Ass-1* KO mice were used in the present study. The results showed that the level of ROS in lung tissue in WT COPD mice was significantly increased as compared with WT control mice. As compared with WT COPD mice, the level of ROS in lung tissue in *Ass-1* KO COPD mice was further increased (Fig. 3J, K). As compared with WT control mice, the level of NO in lung tissue in WT COPD mice was significantly decreased. As compared with WT COPD mice, the level of NO in lung tissue in *Ass-1* KO COPD mice was further decreased.

#### *Ass-1* KO aggravated pathological changes of lung tissue

As for HE staining, the alveoli of WT control mice were intact, and there were only few inflammatory cells infiltrated in interstitial of lung. While, in WT COPD mice, alveolar vacuolation and inflammatory cell infiltration in lung bronchus appeared obvious. As compared with WT COPD mice, in *Ass-1* KO COPD mice, there were a lot of vacuoles in alveoli and a lot of inflammatory cells infiltration in bronchi (Fig. 3L).

As for Masson staining, the results showed that a small amount of collagen fibers was deposited around the lung bronchus in WT control mice. As compared with the WT control group, more collagen fibers were deposited around the lung bronchus in and collagen I contents was increased WT COPD and *Ass-1* KO COPD mice, *Ass-1* KO COPD mice had the most (Fig. 3M, N).

### With BECS

#### CSE decreased cell viability of BECs

**Fig. 4:**
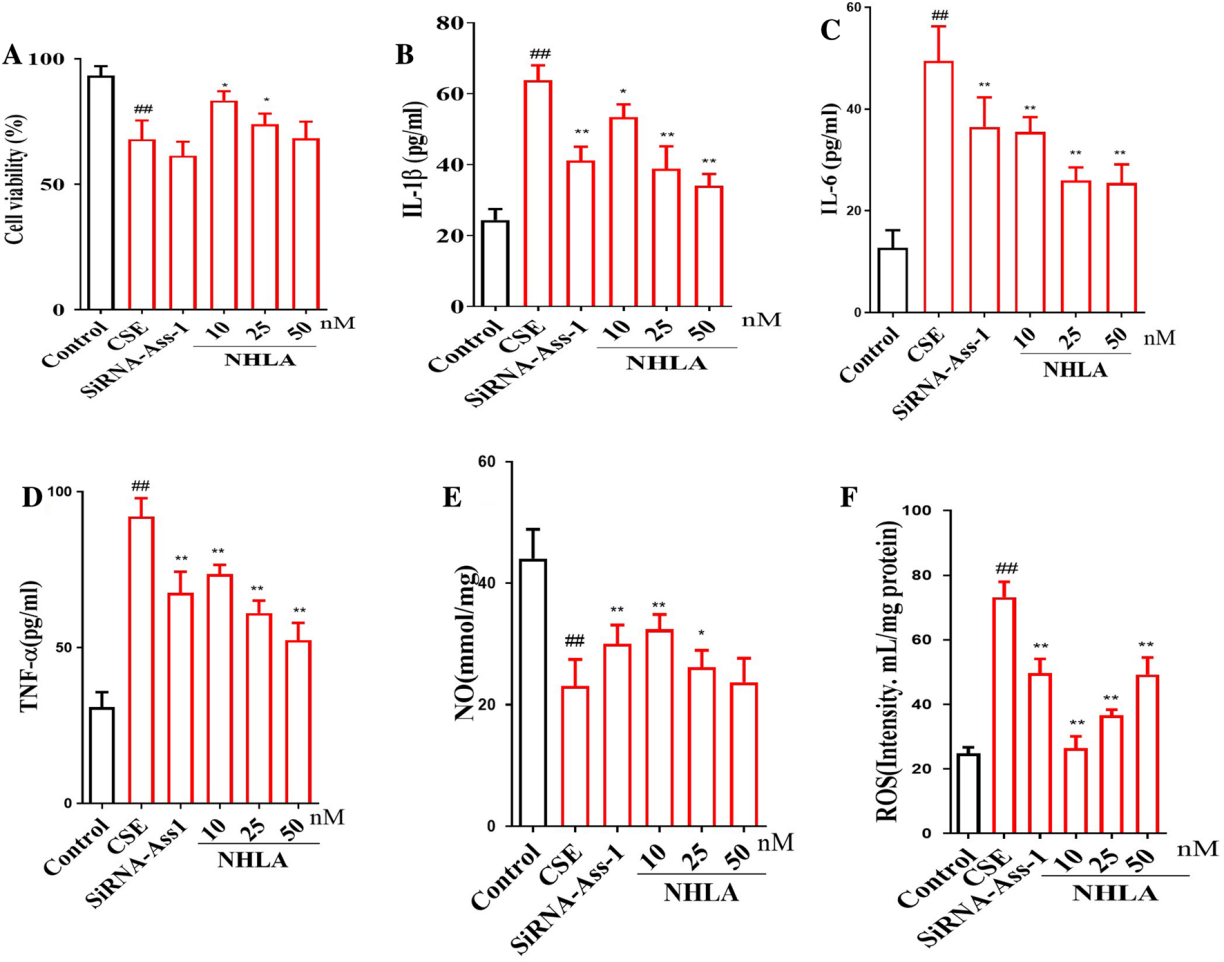
Effects of l-arginine (LA) on cigarette smoke extract (CSE)-induced primary bronchial epithelial cells (BEC) injury. Cell viability (**A**). Interleukin-1β (IL-1β) (**B**), interleukin-6 (IL-6) (**C**) and tumor necrosis factor-α (TNF-α) (**D**) in cell supernatant of CSE-induced BESs. Reactive oxygen species (ROS) levels in CSE-induced BESs (**E**). Nitric oxide (NO) (**F**) content in CSE-induced BESs. (n = 10). All the data were presented as mean ± SD. Compared with control: ^##^P < 0.01

In order to further prove the role of LA in COPD, the primary bronchial epithelial cells (BECs) were used to establish an COPD cell model in vitro. Firstly, the cell vitality was detected. The result showed that CSE significantly decreased the cell viability of BECs as compare with the control group. As compared with CSE group, silence of *Ass-1* further decreased the cell viability of BECs. Meanwhile, NHLA also significantly decreased the cell viability of BECs (Fig. 4A).

#### CSE increased IL-1β, IL-6 and TNF-α in BECs supernatant

In order to verify the role of LA in inflammatory response of COPD. BECs and *Ass-1* KO BECs were used in the study. The results showed that as compared with the control group, CSE stimulation significantly increased the contents of IL-1β, IL-6 and TNF-α in BECs supernatant. Silence of *Ass-1* further increased the contents of IL-1β, IL-6 and TNF-α in BECs. Meanwhile, *Ass-1* inhibitor NHLA also significantly increased the contents of IL-1β, IL-6 and TNF-α in BECs supernatant (Fig. 4B–D).

#### CSE increased ROS and decreased NO in BECs

In order to verify the effect of LA on ROS and NO. BECs and *Ass-1* KO mice, BECs were used in the study. The results showed that as compared with the control group, CSE stimulation significantly increased the level of ROS in BECs, silence of *Ass-1* further increased the level of ROS in BECs. Meanwhile, NHLA also significantly increased the level of ROS in BEC cell. CSE stimulation significantly decreased the level of NO in BECs as compares with the control group, silence of *Ass-1* further decreased the level of NO in BECs. Meanwhile, NHLA also significantly decreased the level of NO in BECs (Fig. 4E, F).

### The underline mechanism of LA in COPD

#### CS increased NLRP3/NF-κB signaling pathway in lung of COPD rats

**Fig. 5:**
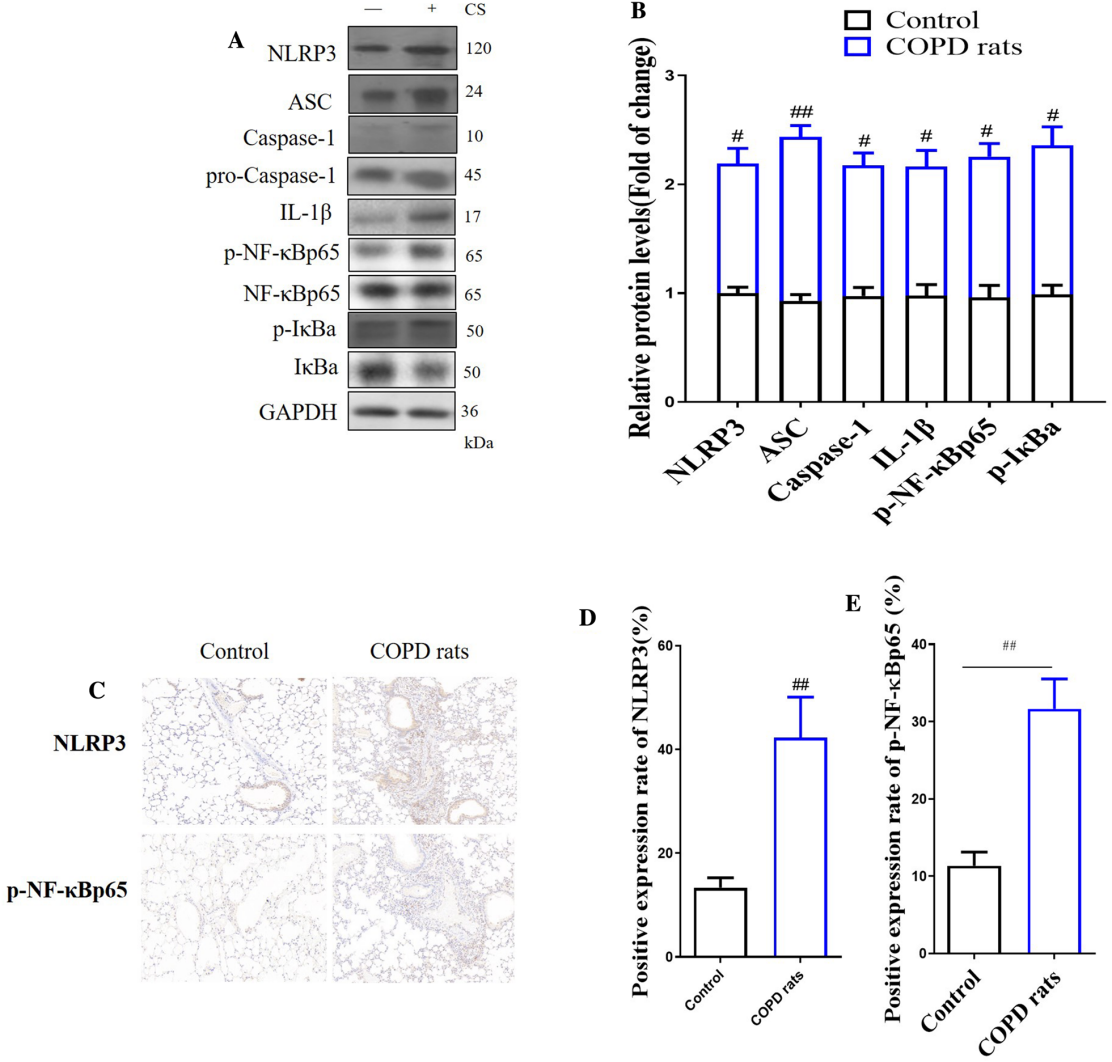
The changes of ROS/NLRP3/NF-κB signaling pathway in COPD rats. Western blot of ROS/NLRP3/NF-κB signaling pathway in lung tissue in COPD rats (**A**), Quantification of ROS/NLRP3/NF-κB signaling pathway in COPD rats (**B**). The expression levels of NLRP3 and p-NF-κBp65 in lung tissue in COPD rats by immunohistochemistry (× 200) (**C**). Quantification of NLRP3 (**D**) and p-NF-κBp65 (**D**) in lung tissue in COPD rats. (n = 3). All data were presented as mean ± SD. Compared with control rats: ^##^P < 0.01

In order to explore the mechanism of LA in COPD, we observed the changes of ROS/NLRP3/NF-κB signaling pathway with COPD rats, so as to preliminarily understand the mechanism of LA in COPD. The results showed that as compared with the control rats, the expression levels of NLRP3, ASC, Caspase-1, IL-1β, p-NF-κBp65 and p-IκBa in the lung tissue of COPD rats were significantly increased (Fig. 5A, B). Immunohistochemistry results showed that the expression of NLRP3 and p-NF-κBp65 in lung tissue of COPD rats were significantly increased as compare with control rats (Fig. 5C–E).

### *Ass-1* KO increased NLRP3/NF-κB signaling pathway in COPD mice

**Fig. 6:**
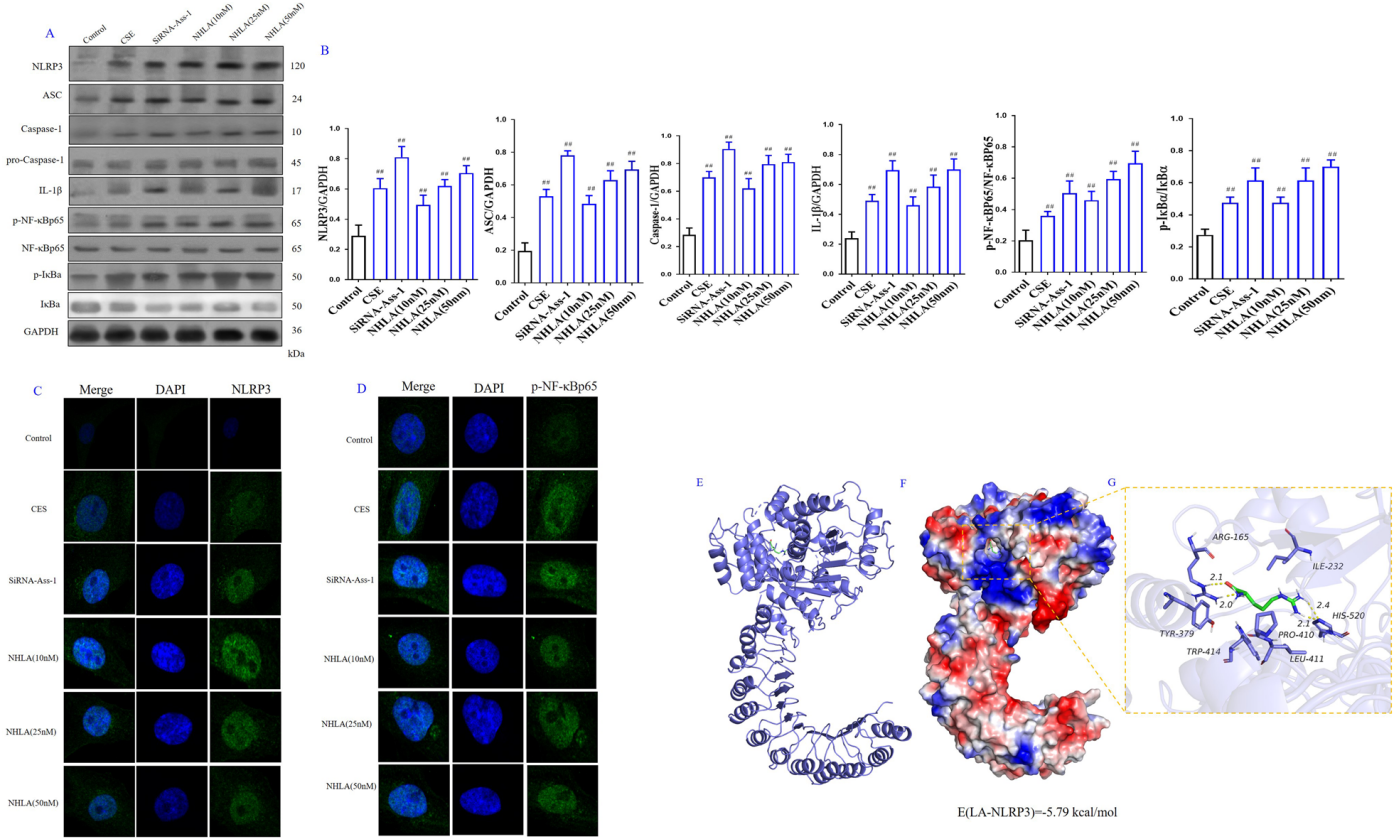
Role of l-arginine (LA) mediated ROS/NLRP3/NF-κB signaling pathway in COPD mice. Western blot of ROS/NLRP3/NF-κB signaling pathway in lung in COPD KO mice (**A**), Quantification of ROS/NLRP3/NF-κB signaling pathway in *Ass-1* KO COPD mice (**B**). The expression levels of NLRP3 and p-NF-κBp65 in lung tissue in *Ass-1* KO COPD mice by immunohistochemistry (× 200) (**C**). Quantification of NLRP3 and p-NF-κBp65 (**D**) of lung tissue in KO COPD mice. (n = 3). All data were presented as mean ± SD. Compared with WT mice: ^##^P < 0.01

In order to elucidate the mechanism of LA protection in COPD, the expression of LA mediated ROS/NLRP3/NF-κB signaling pathway were detected with *Ass-1* KO mice in the present study. The results showed that the expression of NLRP3, ASC, Caspase-1, IL-1β, p-NF-κBp65 and p-IκBa in lung tissues of WT COPD mice was significantly increased as compared with WT mice. The expression of NLRP3, ASC, Caspase-1, IL-1β, p-NF-κBp65 and p-IκBa in lung tissues of *Ass-1* KO COPD mice was further increased as compared with WT COPD mice (Fig. 6A, B). Immunohistochemistry results also showed the expression of NLRP3 and p-NF-κBp65 in lung tissues of WT COPD mice were significantly increased as compared with WT control mice. The expression of NLRP3 and p-NF-κBp65 in lung tissues of *Ass-1* KO COPD mice were further increased as compared with WT COPD mice (Fig. 6C, D).

#### Silence of *Ass-1 and Ass-1* inhibitor, NHLA increased NLRP3/NF-κB signaling pathway in BECs

**Fig. 7:**
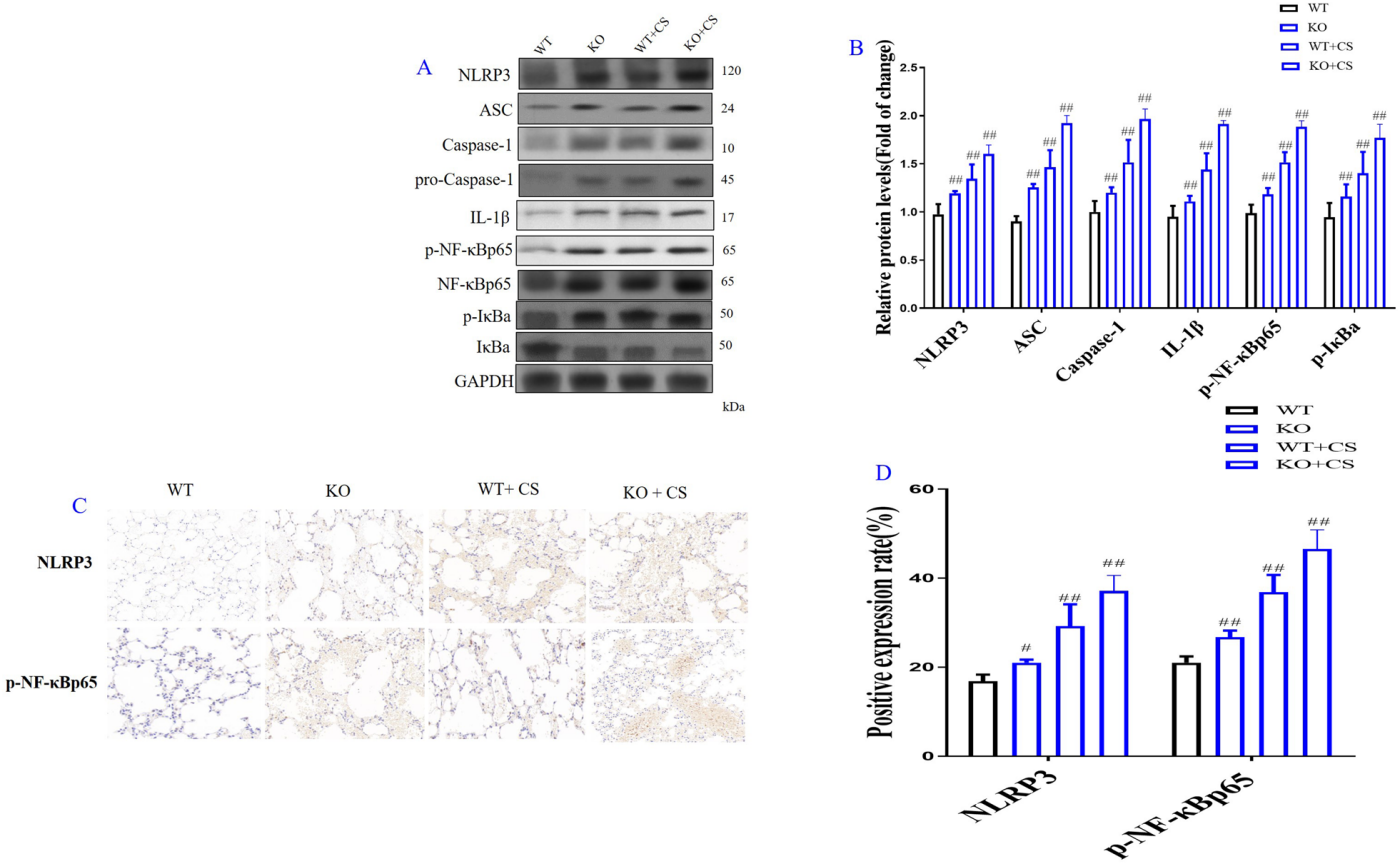
Role of l-arginine (LA) mediated ROS/NLRP3/NF-κB signaling pathway in cigarette smoke extract (CSE)-induced primary bronchial epithelial cell (BEC) injury and molecular docking of LA and NLRP3. Western blot of ROS/NLRP3/NF-κB signaling pathway in CES-induced BECs (**A**), Quantification of ROS/NLRP3/NF-κB signaling pathway in CES-induced BECs (**B**). The expression levels of NLRP3 (**C**) and p-NF-κBp65 (**D**) in CES-induced BECs by immunofluorescence (× 100). (n = 3). Molecular docking result of LA and NLRP3 (**E**): The binding energy predicted by Autodock is − 5.79 kcal/mol for LA-NLRP3 (The binding energy predicted by Autodock <  − 6.00 is considered to be high degree of integration). All data were presented as mean ± SD. Compared with control: ^##^P < 0.01

In order to further verify the role of ROS/NLRP3/NF-κB signaling pathway in the protective effect of LA in COPD, CSE induced BEC injury model and *Ass-1* silence BEC model were used in the present study. The results showed that CSE stimulation significantly increased the expression of NLRP3, ASC, Caspase-1, IL-1β, p-NF-κBp65 and p-IκBa in BECs as compared with the control group. Silence of *Ass-1* further increased the expression of NLRP3, ASC, Caspase-1, IL-1β, p-NF-κBp65 and p-IκBa in BECs as compare with CSE group. Meanwhile, *Ass-1* inhibitor, NHLA also significantly increased the expression of NLRP3, ASC, Caspase-1, IL-1β, p-NF-κBp65 and p-IκBa in BECs (Fig. 7A, B). The immunofluorescence results showed that CSE stimulation significantly increased the expression of NLRP3 and p-NF-κBp65 in BECs as compared with the control group. Silence of *Ass-1* further increased the expression of NLRP3 and p-NF-κBp65 in BECs. Meanwhile, *Ass-1* inhibitor, NHLA also significantly increased the levels of NLRP3 and p-NF-κBp65 in BECs (Fig. 7C, D).

### LA has strong binding affinity to NLRP3

The result showed that LA docked into the cavity of protein NLRP3 with a reasonable fit. The binding energy of LA-NLRP3 predicted by Autodock is − 5.79 kcal/mol. In order to confirm the direct binding of LA and NLRP3, the surface plasmon on resonance technology (SPRT) was adopted. LA exhibited strong binding affinity for NLRP3 with an estimated equilibrium dissociation constant of 5.6 μmol/L (Fig. 7E). The result suggested that LA has direct action on NLRP3.

### The protective effects of exogenous LA (e-LA) on COPD mice and CSE-treated BECs injury model

#### e-LA decreased ROS, IL-1β, IL-6 and TNF-α and increased the content of NO and increased NLRP3/NF-κB signaling pathway in COPD mice

**Fig. 8:**
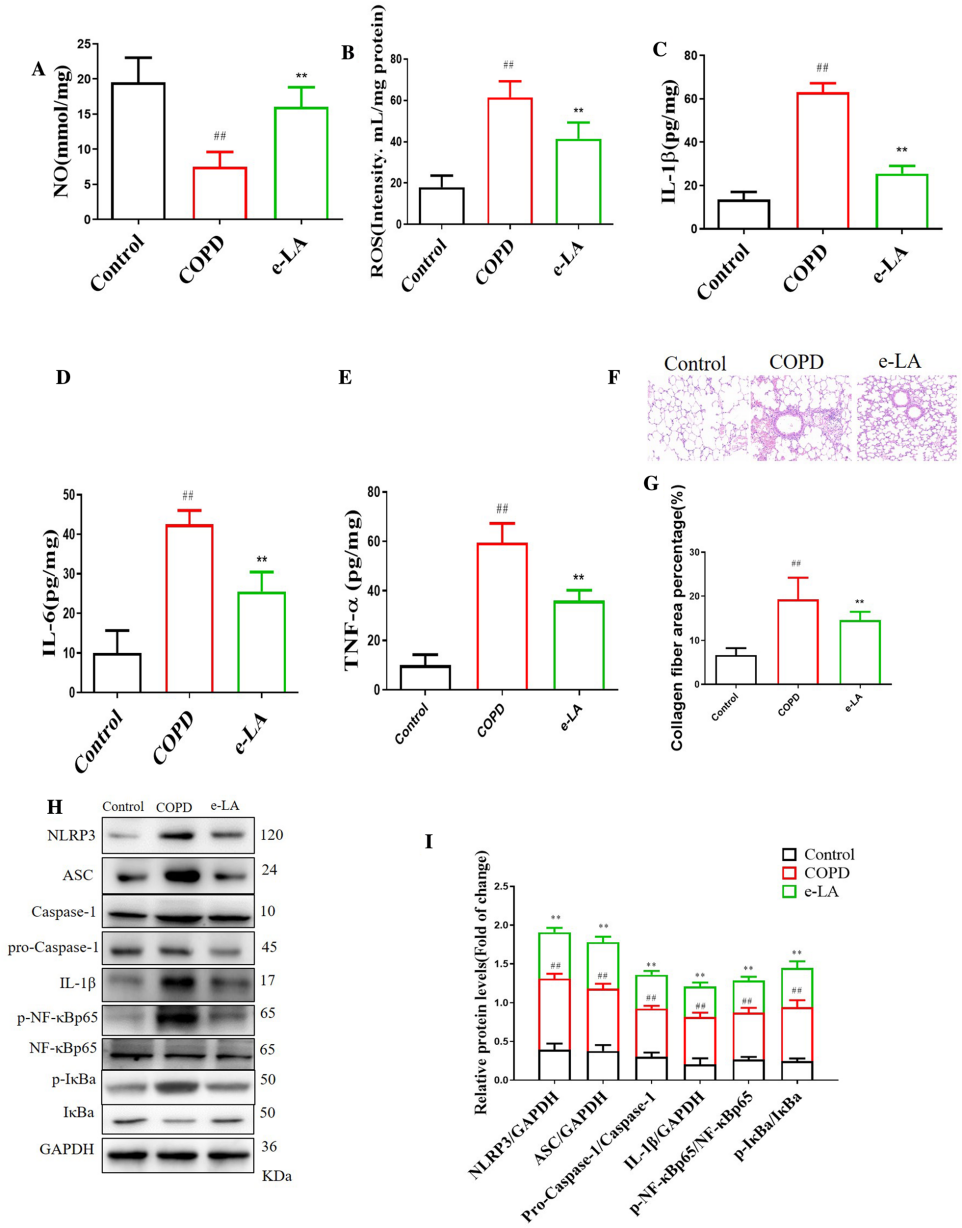
The protective effects of exogenous LA (e-LA) on COPD mice. The effect of e-LA on NO (**A**), ROS (**B**), IL-1β (**C**), IL-6 (**D**) and TNF-α (**E**). HE staining and pathological scores (**F**, **G**). Western blot of ROS/NLRP3/NF-κB signaling pathway in lung of COPD mice (**H**). Quantification of ROS/NLRP3/NF-κB signaling pathway (**I**). (n = 3). All data were presented as mean ± SD. Compared with control: ^##^P < 0.01

Compared with control mice, the levels of ROS, IL-1β, IL-6 and TNF-α were increased and the content of NO was decreased in COPD mice. Compared with COPD mice, e-LA significantly increased the content of NO and decreased the levels of ROS, IL-1β, IL-6 and TNF-α (Fig. 8A–E). HE staining result showed that alveolar vacuolation and inflammatory cell infiltration in lung bronchus appeared obvious in COPD compared with control mice, e-LA significantly restored the above changes (Fig. 8F, G). In terms of mechanism, compared with control mice, the levels of NLRP3, ASC, Caspase-1, IL-1β, p-NF-κBp65 and p-IκBa were increased in COPD mice. Compared with COPD mice, e-LA significantly decreased the levels of NLRP3, ASC, Caspase-1, IL-1β, p-NF-κBp65 and p-IκBa (Fig. 8H, I).

#### e-LA decreased ROS, IL-1β, IL-6 and TNF-α and increased, the content of NO and increased NLRP3/NF-κB signaling pathway in CSE-induced BECs

**Fig. 9:**
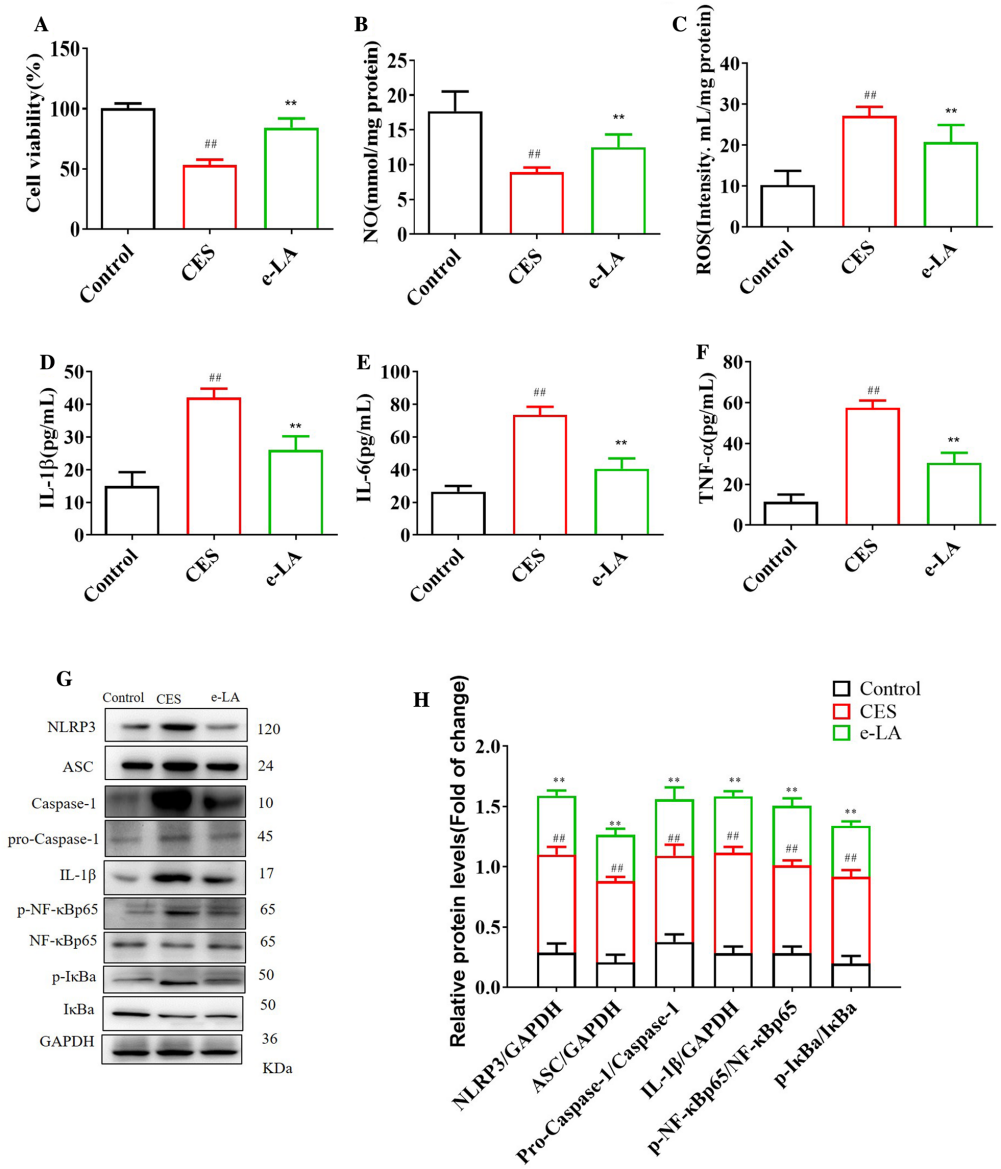
The protective effects of exogenous LA (e-LA) on CSE-induced BECs. The effect of e-LA on cell viability (**A**), NO (**B**), ROS (**C**), IL-1β (**D**), IL-6 (**E**) and TNF-α (**F**). Western blot of ROS/NLRP3/NF-κB signaling pathway in CSE-induced BECs (**G**). Quantification of ROS/NLRP3/NF-κB signaling pathway (**H**). (n = 3). All data were presented as mean ± SD. Compared with control: ^##^P < 0.01

Compared with control, the cell viability was markedly decreased in CSE-induced BECs group, e-LA significantly increased the cell viability (Fig. 9A). Compared with control, the levels of ROS, IL-1β, IL-6 and TNF-α were increased and the content of NO was decreased in CSE-induced BECs model. Compared with COPD mice, e-LA significantly increased the content of NO and decreased the levels of ROS, IL-1β, IL-6 and TNF-α (Fig. 8B–E). In terms of mechanism, compared with control group, the levels of NLRP3, ASC, Caspase-1, IL-1β, p-NF-κBp65 and p-IκBa were increased in CSE-induced BECs model. Compared with CSE-induced BECs, e-LA significantly decreased the levels of NLRP3, ASC, Caspase-1, IL-1β, p-NF-κBp65 and p-IκBa (Fig. 8F–G).

## Discussion

The key metabolite LA was identified and its biological function and mechanisms in COPD were investigated by in vivo and in vitro experiments. it was found that: (1) 85 differential metabolites including LA were identified in COPD patient plasma. (2) LA is an important biomarker in COPD, and as a signal molecule, LA has protective effect on COPD in vivo and in vitro. (3) The effects of LA for COPD are related to its inhibition of ROS/NLRP3/NF-κB signaling pathway, silence of *Ass-1* and *Ass-1* inhibitor, canceled the protective effect of LA on COPD. (4) Exogenous LA also has protective effect, and its mechanism is related to regulating ROS/NLRP 3/NF-κ B signaling pathway.

COPD is a common respiratory system disease, which has severe damage of airway and lung parenchyma. It is believed that the COPD injury is manly related to inflammation, imbalance of protease/anti-protease system and excessive oxidative stress [27]. Because of its high disability and death rate, it seriously affects people's health and the quality of life, so its prevention and treatment are particularly important. The treatments of COPD are mostly limited to symptomatic treatment such as anti-phlegm, anti-cough and anti-asthma, the available treatment drugs are mostly confined to glucocorticoids and antibiotics [28]. These treatment measures are not only non-lasting, but also have great side effects. For example, for some elderly patients, long-term application of β2 adrenergic receptor agonist and aerosol inhalation of anticholinergic drugs may lead to glaucoma. Long-term use of glucocorticoids may cause immunosuppression. Therefore, many researchers have long been devoted themselves to finding effective COPD treatment drugs, but the results are not ideal. Therefore, in recent years, many researchers are making great efforts to search for the new targets of COPD.

Biomarkers have irreplaceable role in the development of diseases and searching for their new treatment targets [29]. Metabonomics technology is one of the beneficial means to find biomarkers and new targets of diseases, which can reveal the metabolites profile, and understand the metabolites types and contents for variety of diseases.Therefore, metabonomics has begun to be gradually applied to the diagnosis and target discovery of various diseases.

In recent years, there have been studies on the metabolism of COPD by using metabonomic technology. For example, Novotna et al. used HPLC–MS/MS method and found 15 potential biomarkers in COPD patient plasma [30]. Ubhi et al. used HPLC–MS/MS and found 13 different metabolites in COPD patient urine, these substances may be the symbolic metabolites of COPD patients [26]. Singh et al. used nuclear magnetic resonance (NMR) technology to study the plasma metabonomic of COPD patients and found abnormal metabolism mechanism of various amino acids [31]. In addition to clinical studies, there were also animal metabonomic studies about COPD. Li et al. used HPLC–MS/MS technology in COPD rat model, and they found 10 potential markers for COPD [32, 33]. These studies were involved in plasma and urine metabonomic of COPD patients and plasma metabonomic of COPD animals. The metabolites were involved in amino acid metabolism, lipid metabolism, carbohydrate metabolism and many other aspects. Our present study used metabonomic technology to analyze the plasma metabolites of COPD patients. 85 differential metabolites were identified from the plasma of COPD patients, these metabolites were mainly concentrated in amino acids and fatty acids. Consistent with previous research results, in our experiment, the metabolites are mainly concentrated in amino acid metabolism, lipid metabolism. However, the involved amino acids and lipid metabolites are different. It is suggested that the study of different metabolites may have some guidance for the selection of potential markers of COPD.

Previous studies mainly focused of the changes of metabolites in COPD, but did not pay attention to the role of metabolites in the prevention and treatment of COPD. In the present study, after obtained the 85 differential metabolites, we adopted the TOP50 analysis and the hot spot analysis to find the largest changeable (decreased most) metabolite among the 85 differential metabolites was LA. It is worth mentioning that the previous literature reports that there was a certain controversy between the concentration changes of plasma LA of COPD compared with healthy people. Ruzsics et al. reported that plasma LA was significantly increased compared with healthy people [34]. While, Jonker et al. reported that plasma LA was significantly decreased compared with healthy people [35]. In our study, plasma LA significantly decreased in COPD patients. We speculate that it may be related to the age and course of COPD. It was previously reported that LA has a close relationship with lung disease. l-Arginine/NO homeostasis has been found to play a role in various conditions affecting lung and lung diseases [such as asthma, chronic obstructive pulmonary disease (COPD), cystic fibrosis (CF), pulmonary hypertension and bronchopulmonary dysplasia [36]. There was a close relationship between arginase-2 and airway inflammation, and arginase-2 deficiency will lead to airway inflammation [37]. In addition, some previous studies were consistent with our research. They reported that LA was an important biomarker of COPD. Istvan et al. reported that the level of LA was significantly increased in patients with COPD [35]. Samuel et al. LA ameliorates cigarette smoke-induced emphysema in mice [38]. This study not only found that LA is an important biomarker of COPD through metabolomics, but also investigated the effect and mechanism of LA on COPD. In this study, with *Ass-1* KO mice, we found LA was significantly decreased. *Ass-1* is a rate-limiting enzyme for synthesizing LA, its KO may reduce LA biosynthesis. LA is the main substrate for endogenous NO. NO is a multifunctional molecule, which plays important role in the physiological and pathological process of respiratory system [39–41]. It has been reported that LA may reduce ROS [42]. ROS may destroy the dynamic balance of cell oxidation and redox response and the function of endoplasmic reticulum. Excessive ROS may induce the imbalance of endogenous protease/antiprotease to accelerate the lung injury. Meanwhile, excessive ROS may lead to DNA damage, lipid peroxidation and carbonylation of protein in airway cells, which may lead to the aggravation of COPD symptoms [43]. Therefore, we speculated that LA may be an important biomarker of COPD and play a protective effect in COPD via reducing ROS, inhibiting inflammatory response and increasing NO production. In order to testify this hypothesis, *Ass-1* KO mice, *Ass-1* silence BECs and *Ass-1* inhibitor, NHLA were adopted in the present study. It was found that *Ass-1* KO, *Ass-1* silence or *Ass-1* inhibitor aggravated the COPD symptoms in COPD mice and increased CSE induced BECs injury. The contents of ROS and IL-1β, IL-6, TNF-α in serum and BALF of COPD mice or BECs supernatant were increased, while the NO contents were decreased. These results suggest that LA may participate in the pathogenesis of COPD, LA is an important biomarker of COPD. But the mechanism of LA in COPD is not clear.

As mentioned above, LA may produce NO and reduce the production of ROS [44]. ROS is the main activation pathway of NLRP3 inflammasome, which played important role in the pathogenesis of COPD [45–47]. NF-κB was the critical transcription factor of many inflammatory factors and also plays important role in COPD [48]. We speculated that LA plays the protective effect on COPD possibly via inhibition of ROS/NLRP3/NF-κB pathway and related signaling molecules. With *Ass-1* KO mice, *Ass-1*silence BECs and *Ass-1* inhibitor, via in vivo and in vitro experiments, we testified the hypothesis and proved that LA playing the protective effect on COPD was via inhibition of ROS/NLRP3/NF-κB pathway. The absence of LA aggravated the symptoms of COPD. Additionally, molecule docking experiment showed LA directly acted on NLRP3. The result suggests that LA participation of the pathogenesis of COPD is except for being related to inhibition of ROS/NLRP3/NF-κB pathway, the direct action with NLRP3 was also an important mechanism. Finally, we also tested the protective effect of exogenous LA on COPD. The results shown exogenous LA also has protective effects on COPD, and its mechanism was related to the regulation of xx signaling pathway.

## Conclusion

L-arginine is a key metabolic marker for COPD patients, which plays important protective effect on COPD. The mechanism is mainly related to ROS/NLRP3/NF-κB signaling pathway. It may be a novel therapeutic target for the treatment of COPD. However, this study has some limitations: (1) For the mechanism study, we did not intervene other key molecules of the signal pathway to prove the target of LA. (2) For COPD animal model, we only used CS-induced COPD model while did not use other COPD model to verify the efficacy of LA. (3) The in-depth mechanisms of LA on COPD need to further be studied.

## Supplementary Information

**Additional File 1: Fig. S1** Typical CT imaging characteristics of COPD patients.

**Additional File 2: Fig. S2** Sequences of Ass-1 siRNAs.

**Additional File 3: Fig. S3** OPLS-DA analysis.

**Additional File 4: Fig. S4** LA detection in serum of COPD patients and KO mice by ELISA.

**Additional File 5: Table S1** Eighty-five differential metabolites identified by UPLC-TOFMS between two groups.

**Additional File 6: Table S2** Quantification of the Top50 different metabolites.

## Data Availability

The data used to support the findings of this study are available from the corresponding author upon request.
