# Correction: l-Arginine, as an essential amino acid, is a potential substitute for treating COPD via regulation of ROS/NLRP3/NF-κB signaling pathway

**DOI:** 10.1186/s13578-025-01373-2

**Published:** 2025-03-17

**Authors:** Chunhua Ma, Kexi Liao, Jing Wang, Tao Li, Liangming Liu

**Affiliations:** 1 State Key Laboratory of Trauma, Burns and Combined Injury, Shock and Tranfusion Research, Department of Army Medical Center, Army Medical University, Chongqing, 400042 People’s Republic of China; 2 The Affiliated Nanjing Hospital of Nanjing University of Chinese Medicine, Nanjing, 210001 China; 3 Institute of Hepatobiliary Surgery, First Affiliated Hospital, Army Medical University, Shapingba District, Gaotanyan Road 30, Chongqing, 400038 China; 4 School of Biology and Food Engineering, Institute of Pharmaceutical Biotechnology, Suzhou University, Anhui, China

**Correction: Cell & Bioscience (2023) 13:152** 10.1186/s13578-023-00994-9

In this article [1], the wrong figures appeared as Figs. 3 and 7; the figures should have appeared as shown below.

Incorrect Fig. 3
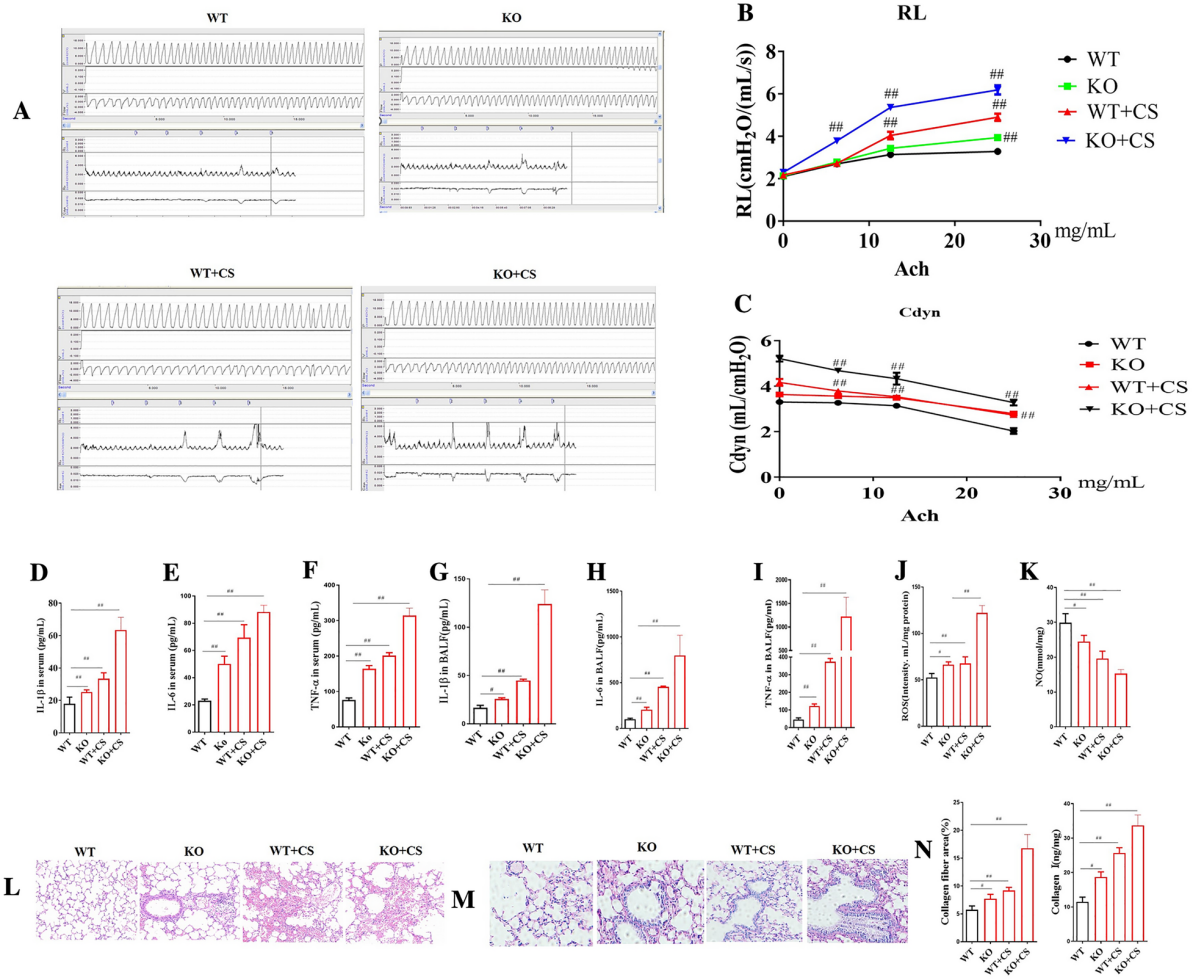


Correct Fig. 3

**Fig. 3:**
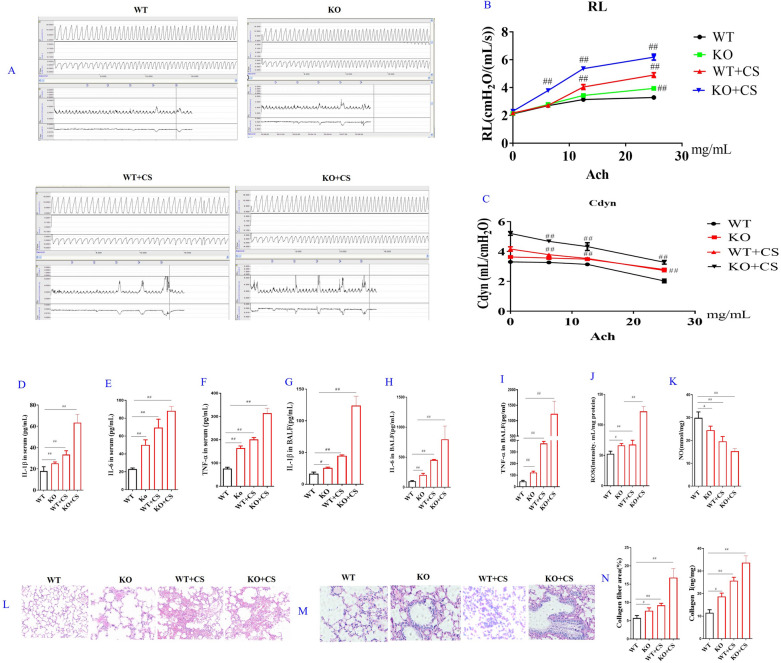
Effects of l-arginine (LA) on KO COPD mice. Airway reaction (**A**). The percentage changes of the resistance of lung (RL) (**B**) and lung dynamic compliance (Cdyn) (**C**) in WT and KO COPD mice. Serum cytokines: The contents of interleukin-1β (IL-1β) (**D**), interleukin-6 (IL-6) (**E**), tumor necrosis factor-α (TNF-α) (**F**). BALF cytokines: the contents of Interleukin-1β (IL-1β) (**G**), interleukin-6 (IL-6) (**H**), tumor necrosis factor-α (TNF-α) (**I**). Reactive oxygen species (ROS) (**J**) and nitric oxide (NO) (**K**) contents in lung tissues. Pathological changes (HE staining) and Masson staining of lung in COPD rats: HE staining of lung in COPD rats (× 200) (**L**), Masson staining of lung in COPD rats (× 200) (**M**), Collagen quantification of Masson staining and collagen I contents of lung in COPD KO mice (**N**). (n = 10). All data were presented as mean ± SD. Compared with WT mice: ^##^P < 0.01

Incorrect Fig. 7
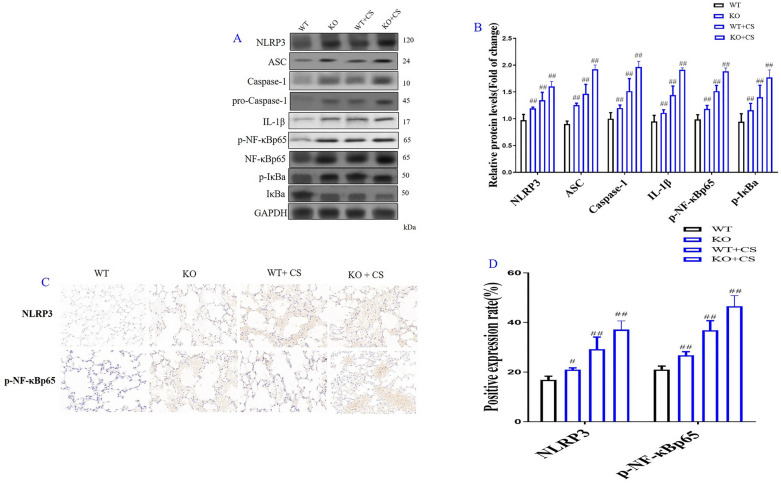


Correct Fig. 7

**Fig. 7:**
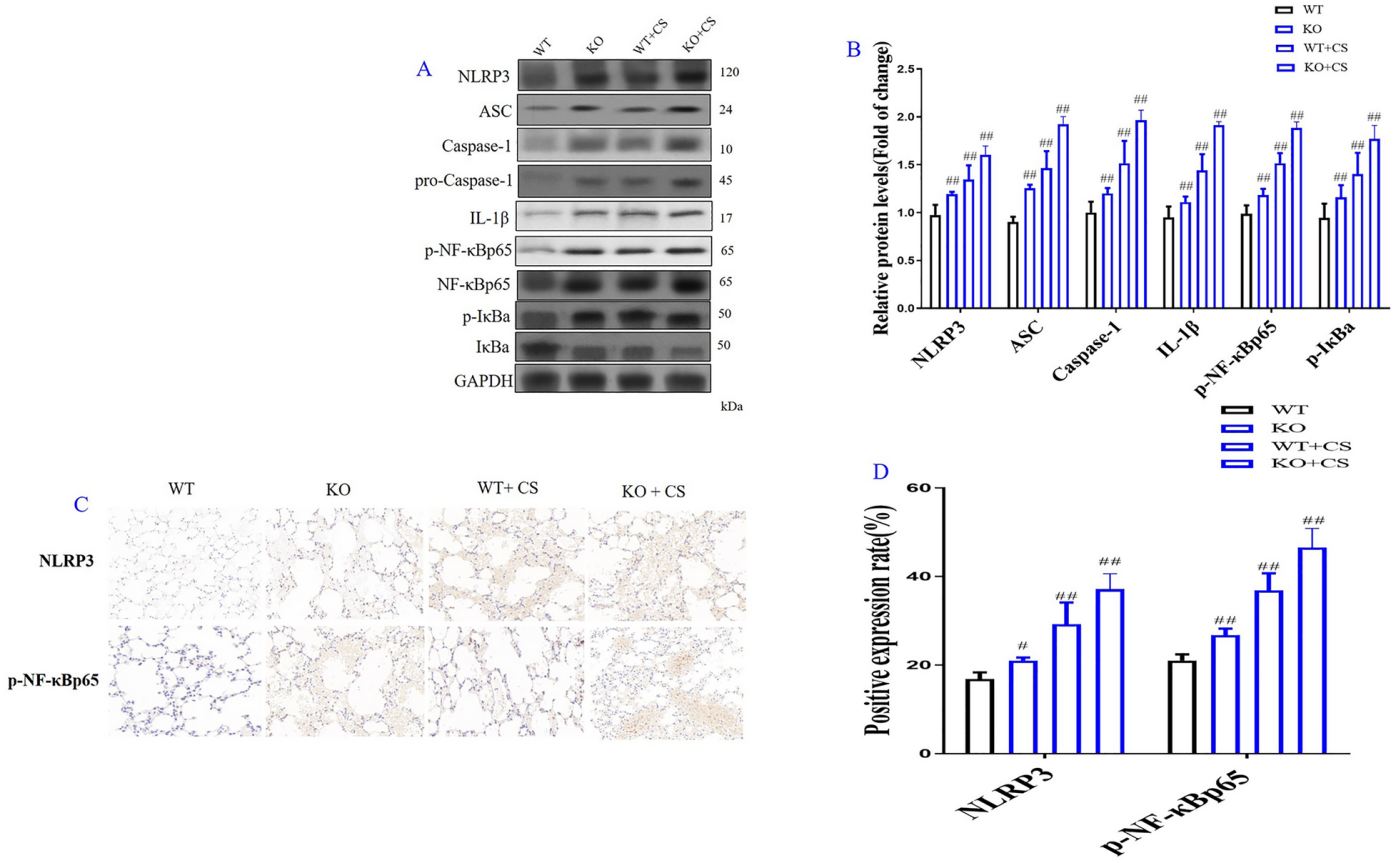
Role of l-arginine (LA) mediated ROS/NLRP3/NF-κB signaling pathway in cigarette smoke extract (CSE)-induced primary bronchial epithelial cell (BEC) injury and molecular docking of LA and NLRP3. Western blot of ROS/NLRP3/NF-κB signaling pathway in CES-induced BECs (**A**), Quantification of ROS/NLRP3/NF-κB signaling pathway in CES-induced BECs (**B**). The expression levels of NLRP3 (**C**) and p-NF-κBp65 (**D**) in CES-induced BECs by immunofluorescence (× 100). (n = 3). Molecular docking result of LA and NLRP3 (**E**): The binding energy predicted by Autodock is − 5.79 kcal/mol for LA-NLRP3 (The binding energy predicted by Autodock <  − 6.00 is considered to be high degree of integration). All data were presented as mean ± SD. Compared with control: ^##^P < 0.01
