# Supplementary material for: l-Arginine, as an essential amino acid, is a potential substitute for treating COPD via regulation of ROS/NLRP3/NF-κB signaling pathway
Source: Cell Biosci. 2023 Aug 18;13:152. doi: 10.1186/s13578-023-00994-9 (PMC10436497; doi:10.1186/s13578-023-00994-9)

**Additional File 1: Fig. S1 Typical CT imaging characteristics of COPD patients**

**
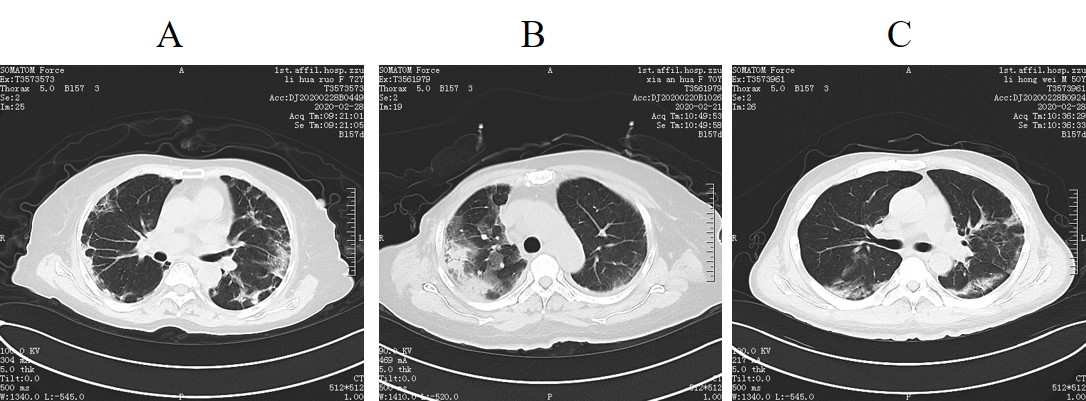
**

A: thickened lung texture, disorder lung texture, twisted;(B) increased lung volume, thinned lung texture and increased lung brightness; (C) thickened bronchial wall.

**Metabolic fingerprints of plasma in COPD patients**

Healthy people in positive ion mode (A), COPD patients in positive ion mode (B), Healthy people in negative ion mode (C), COPD patients in negative ion mode (D).

A

B

C

D

**Identification of bronchial epithelial cells (BESs)**

**Immunofluorescence staining of keratin CK14 in BESs**

The levels of CK14 in BECs were evaluated by immunofluorescence. Briefly, cultured BECs were washed twice with PBS, fixed with 4% paraformaldehyde (PFA) for 30 min, and then permeabilized with 0.5% Triton X-100 in PBS for 5 min, blocked with 5% BSA for 1h. The cells were incubated with the primary antibodie a-SMA (1:500) overnight at 4℃, washed three times with PBS and incubated with goat anti-rabbit IgG (H+L) secondary antibody, Alexa Fluor® 488 conjugate (1:500) for 1h. After three times washing with PBS and the DAPI was done at room temperature for 5 min. Fluorescence images were taken with fluorescence microscopy.

The first day:

**
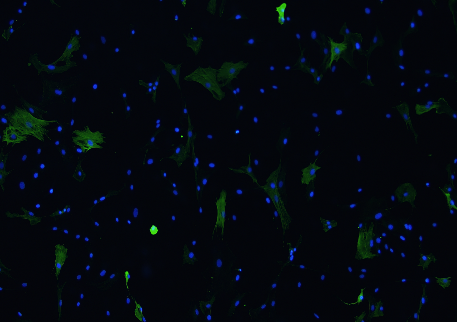
**

The second days:


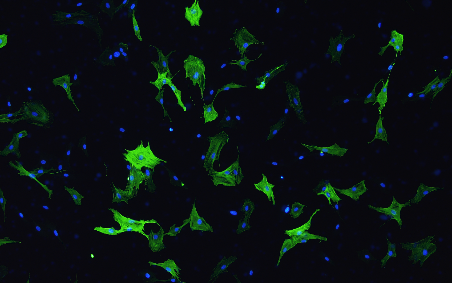


The third day


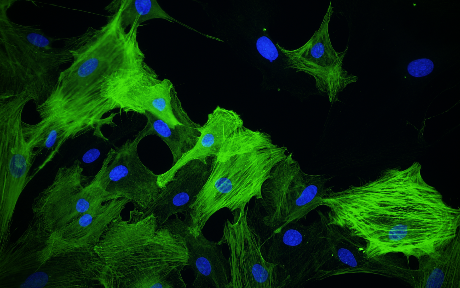

Supplement: Supplementary file 1 — Additional File 1: Fig. S1 Typical CT imaging characteristics of COPD patients. [file 13578_2023_994_MOESM1_ESM.docx]
