# Supplementary material for: l-Arginine, as an essential amino acid, is a potential substitute for treating COPD via regulation of ROS/NLRP3/NF-κB signaling pathway
Source: Cell Biosci. 2023 Aug 18;13:152. doi: 10.1186/s13578-023-00994-9 (PMC10436497; doi:10.1186/s13578-023-00994-9)
Supplement: Supplementary file 2 — Additional File 2: Fig. S2 Sequences of Ass-1 siRNAs. [file 13578_2023_994_MOESM2_ESM.docx]

**Additional File 2: Fig. S2 Sequences of Ass-1 siRNAs**

**5’-TTATAACCTGGGATGGGCACC-3’**

**5’-TGGACATAGCGTCTGGGATTG-3’**
