# Supplementary material for: l-Arginine, as an essential amino acid, is a potential substitute for treating COPD via regulation of ROS/NLRP3/NF-κB signaling pathway
Source: Cell Biosci. 2023 Aug 18;13:152. doi: 10.1186/s13578-023-00994-9 (PMC10436497; doi:10.1186/s13578-023-00994-9)

**Additional File 3: Fig. S3 Bioinformatics analysis by OPLS-DA analysis, OPLS-DA analysis, PLS-DA analysis and permutation analysis**

OPLS-DA analysis


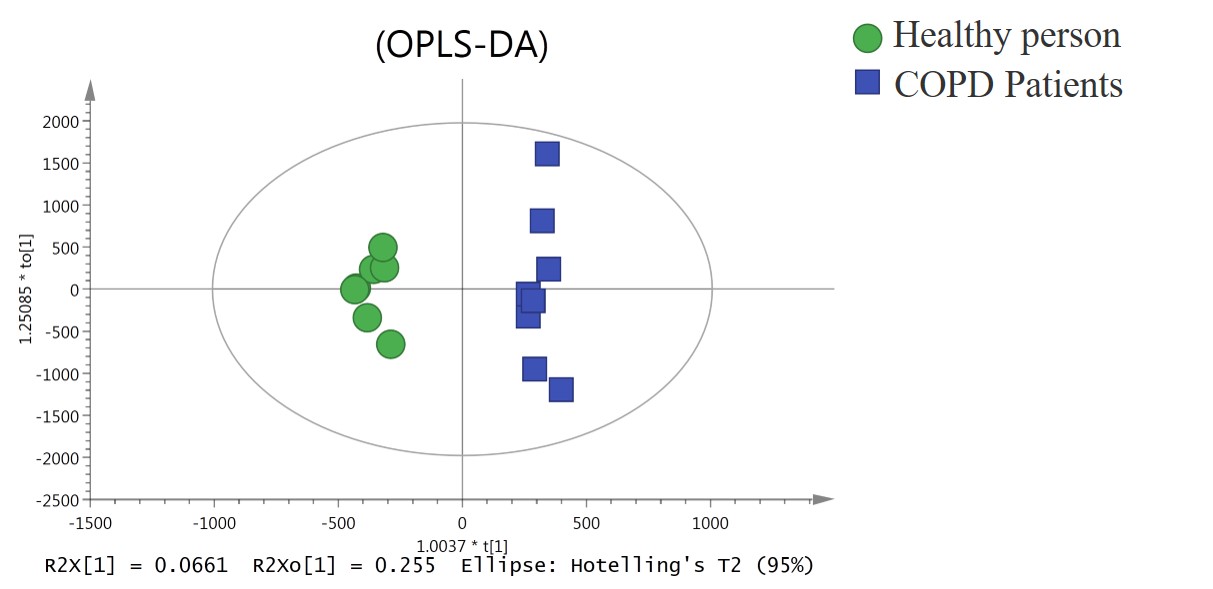


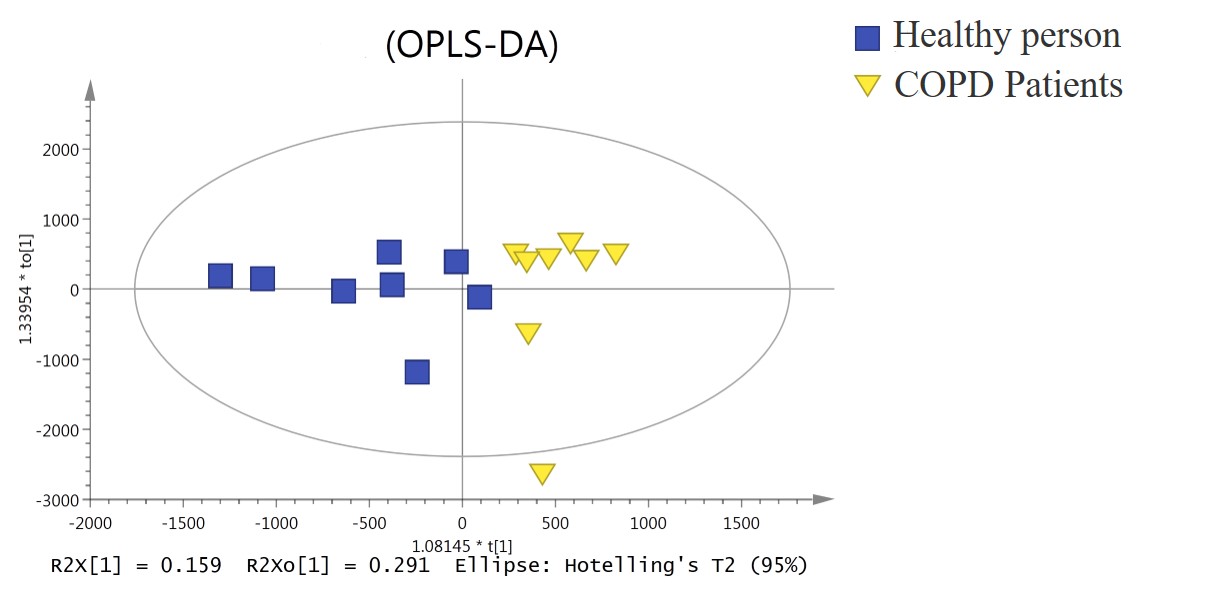


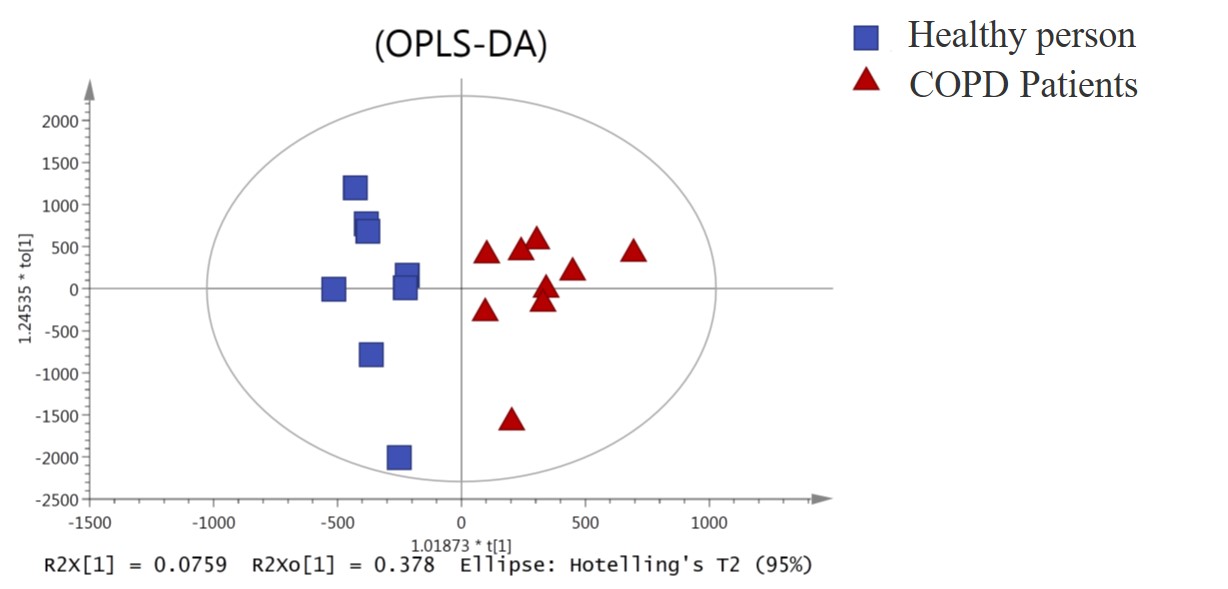


OPLS-DA analysis


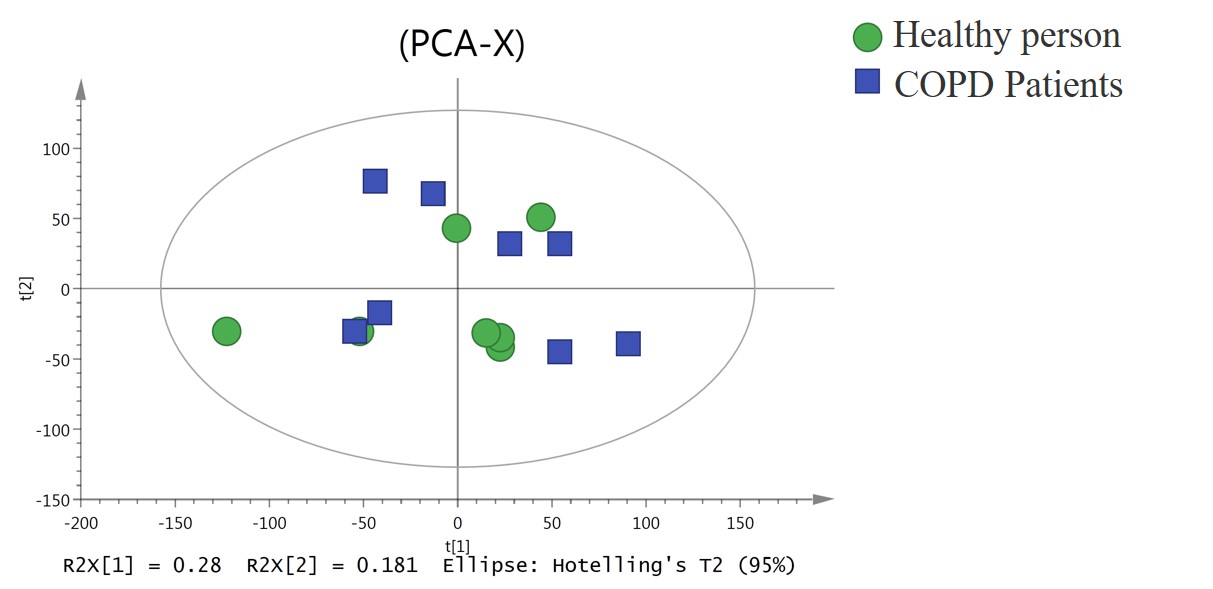


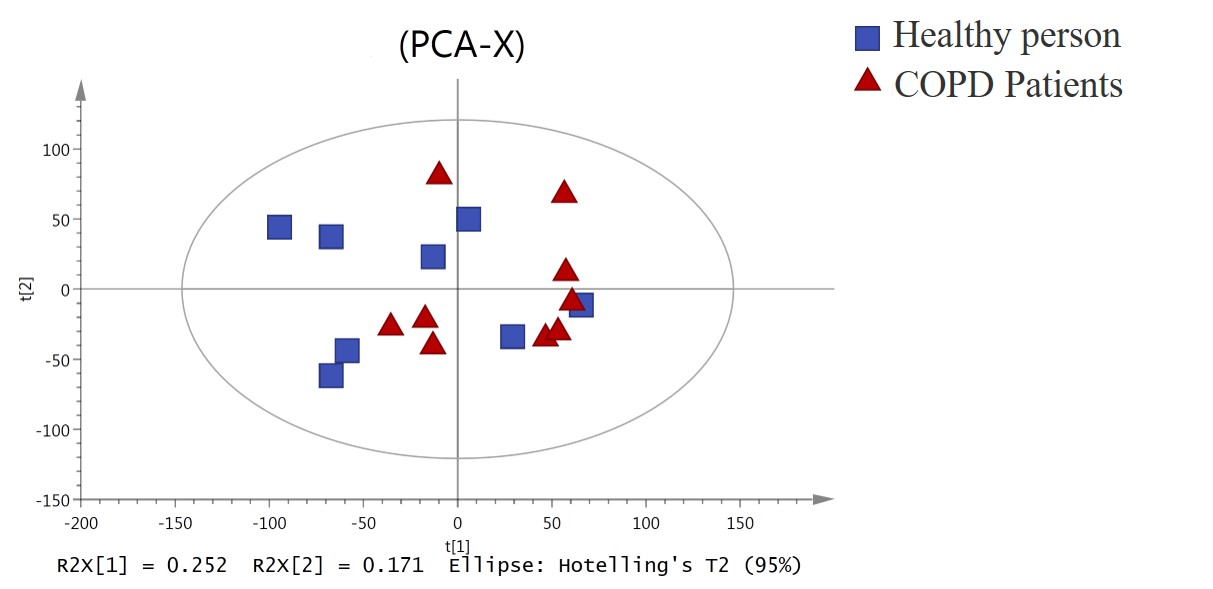


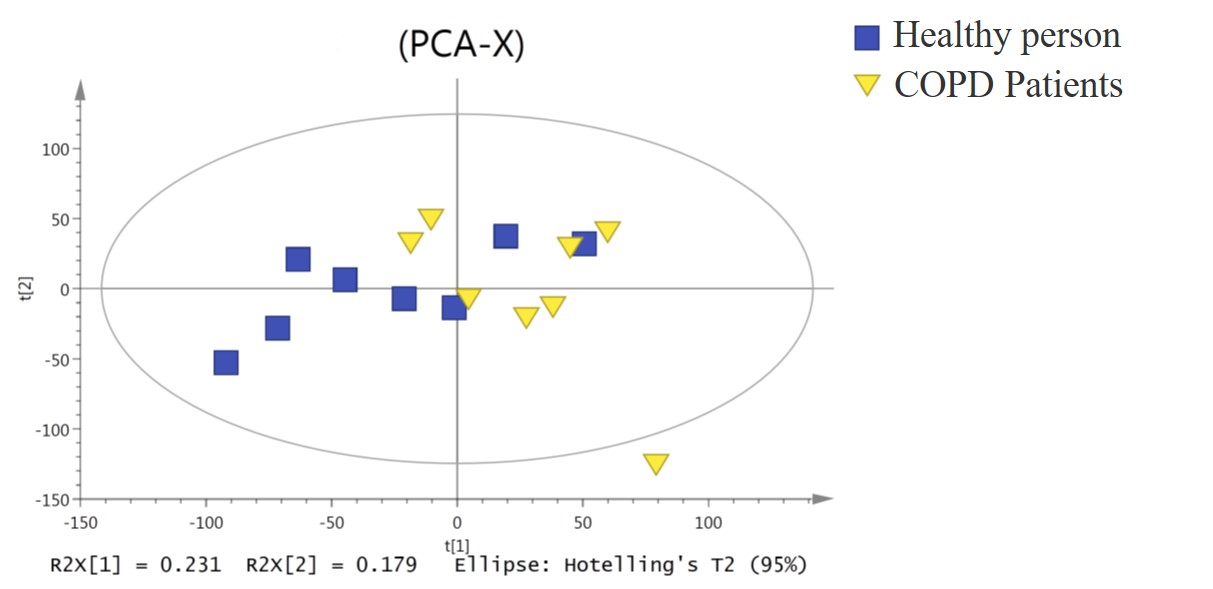


PLS-DA analysis


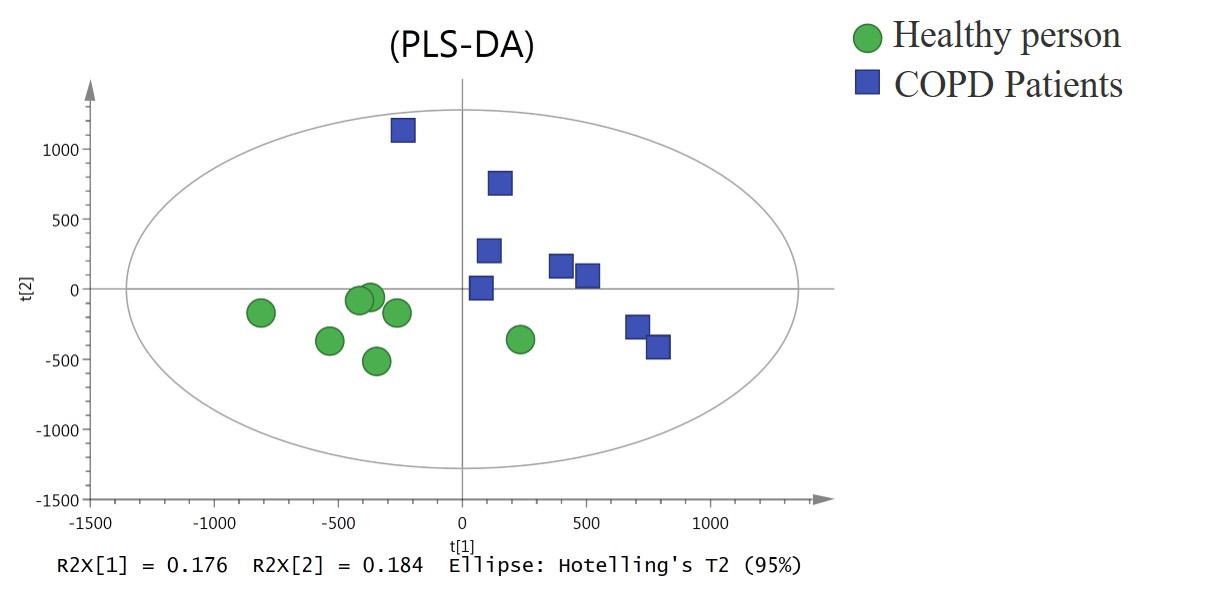


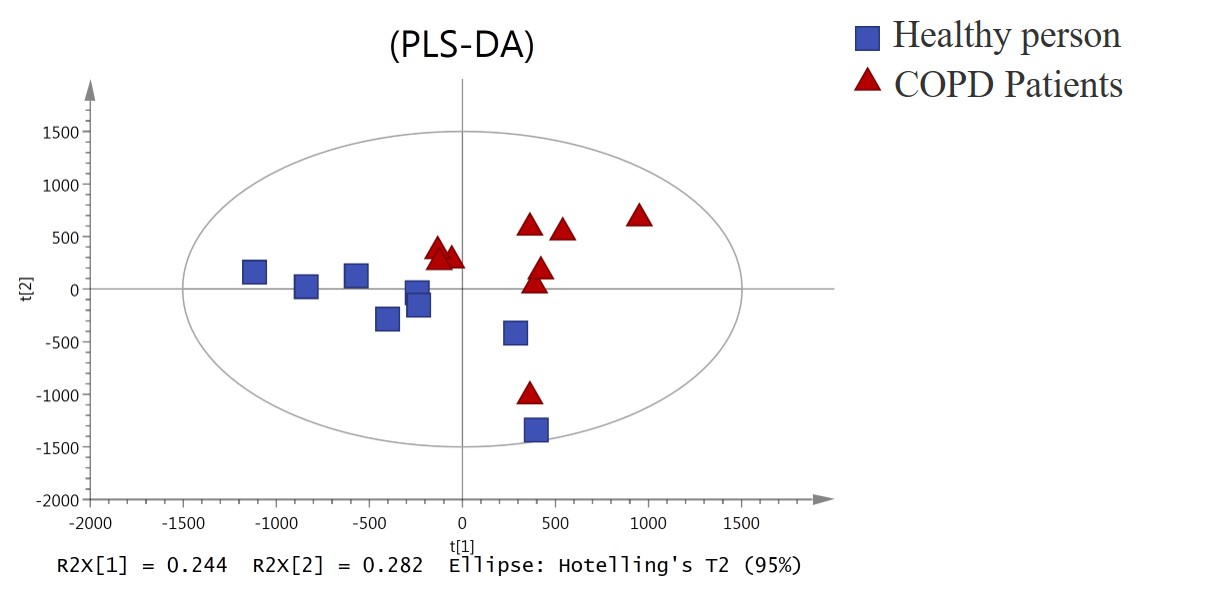


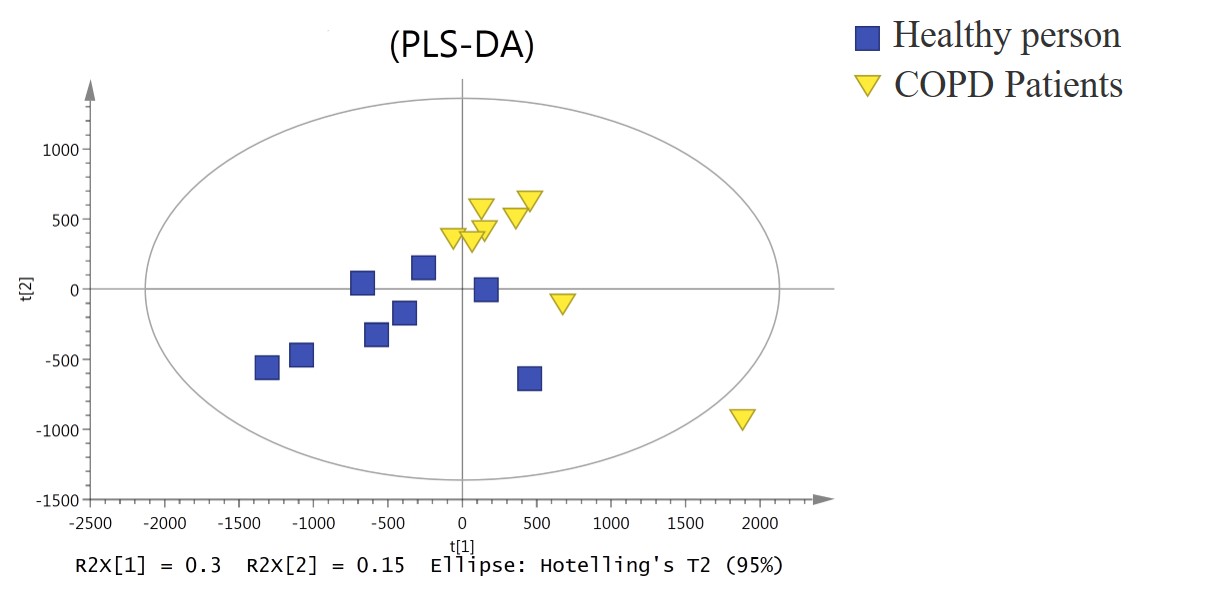


Permutation analysis


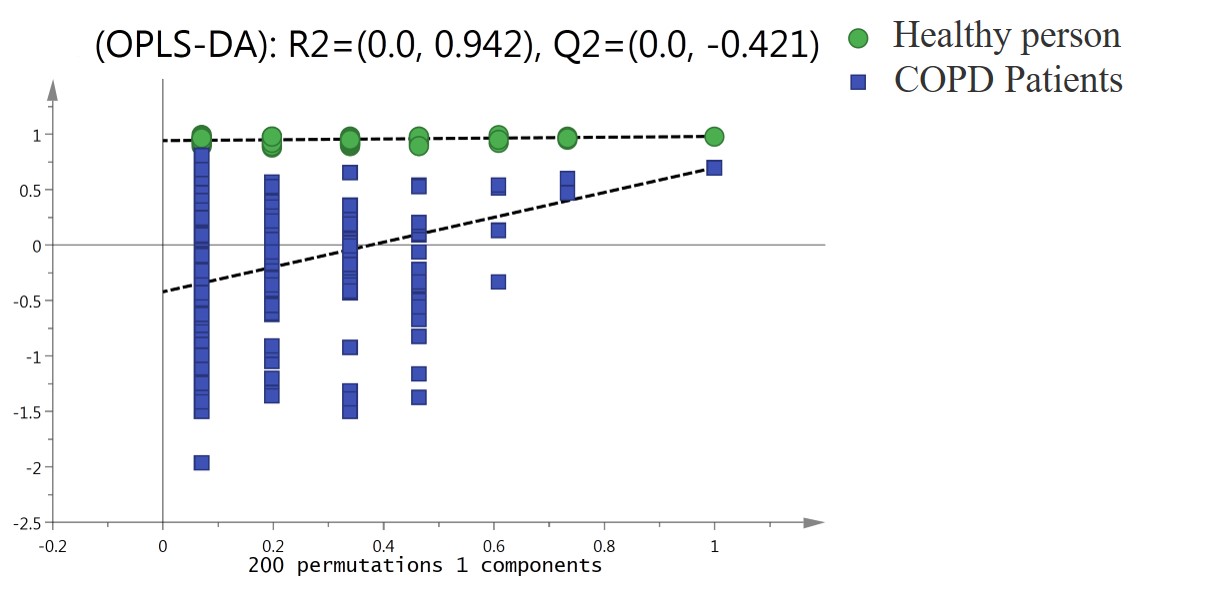


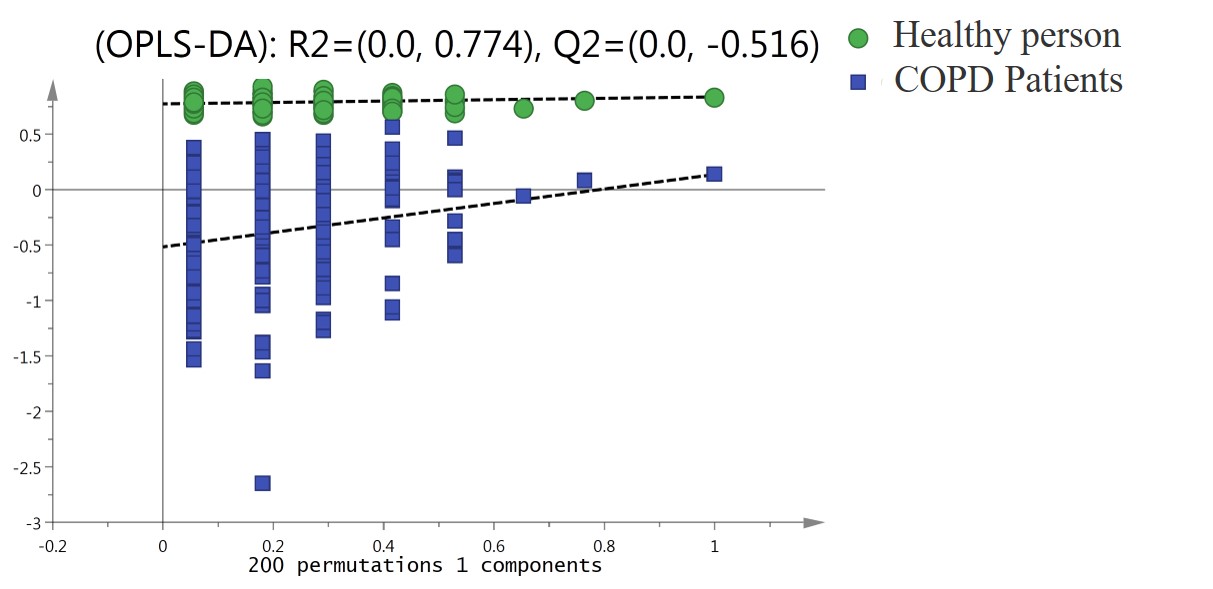


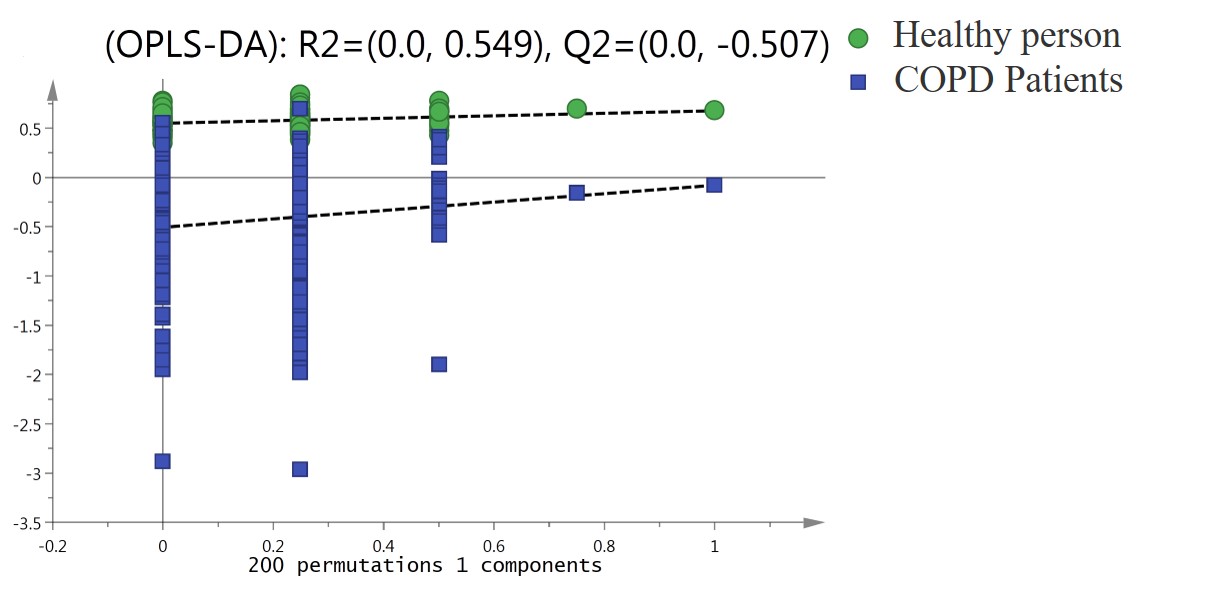

Supplement: Supplementary file 3 — Additional File 3: Fig. S3 OPLS-DA analysis. [file 13578_2023_994_MOESM3_ESM.docx]
