# Supplementary material for: l-Arginine, as an essential amino acid, is a potential substitute for treating COPD via regulation of ROS/NLRP3/NF-κB signaling pathway
Source: Cell Biosci. 2023 Aug 18;13:152. doi: 10.1186/s13578-023-00994-9 (PMC10436497; doi:10.1186/s13578-023-00994-9)

**Additional File 4: Fig. S4 LA detection in serum of COPD patients and KO mice by ELISA**

Enzyme-linked immunosorbent assay (ELISA) was used to detect the contents of LA in serum of COPD patients (A) and mice (B). All the operation steps were strictly performed in accordance with the instructions of ELISA kits.


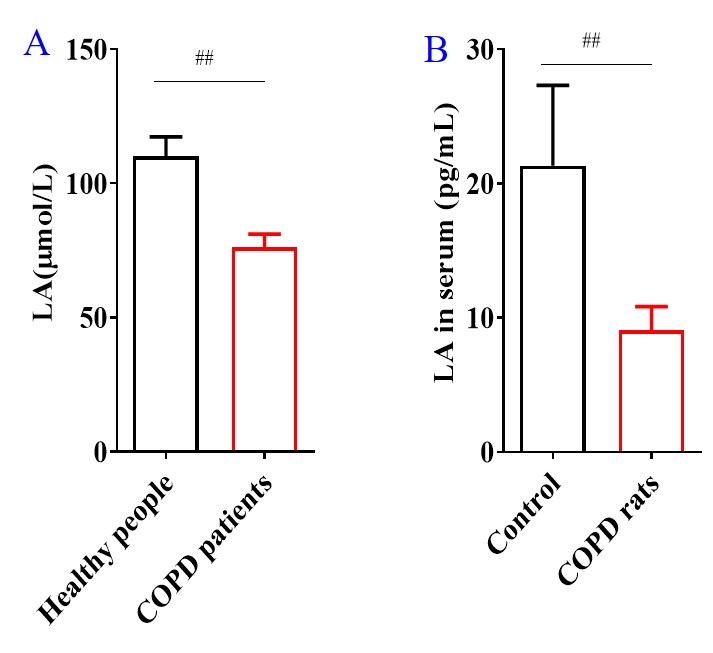

Supplement: Supplementary file 4 — Additional File 4: Fig. S4 LA detection in serum of COPD patients and KO mice by ELISA. [file 13578_2023_994_MOESM4_ESM.docx]
