# Supplementary material for: l-Arginine, as an essential amino acid, is a potential substitute for treating COPD via regulation of ROS/NLRP3/NF-κB signaling pathway
Source: Cell Biosci. 2023 Aug 18;13:152. doi: 10.1186/s13578-023-00994-9 (PMC10436497; doi:10.1186/s13578-023-00994-9)
Supplement: Supplementary file 5 — Additional File 5: Table S1 Eighty-five differential metabolites identified by UPLC-TOFMS between two groups. [file 13578_2023_994_MOESM5_ESM.docx]

**Additional File 5: Table S1 Eighty-five differential metabolites identified by UPLC-TOFMS between two groups**

| NO. | Identification | *m/z* | Retention Time, min |
| --- | --- | --- | --- |
| 1 | L-Arginine | 173.988 | 0.653 |
| 2 | Jimenezin | 605.477 | 10.406 |
| 3 | PG (13:0/20:4(5Z,8Z,11Z,14Z)) | 751.452 | 10.505 |
| 4 | PG(P-20:0/19:1(9Z)) | 841.575 | 10.666 |
| 5 | PS (18:4(6Z,9Z,12Z,15Z)/19:1(9Z)) | 778.501 | 10.826 |
| 6 | PS (18:0/18:2(9Z,12Z)) | 788.544 | 10.929 |
| 7 | PE (13:0/22:0) | 778.561 | 11.071 |
| 8 | 19'-Hexanoyloxymytiloxanthin | 735.456 | 11.102 |
| 9 | (+/-)14,15-EpETrE-(d8) | 373.275 | 11.169 |
| 10 | PE (15:0/20:0) | 732.553 | 11.206 |
| 11 | PS (15:1(9Z)/22:4(7Z,10Z,13Z,16Z)) | 778.499 | 11.358 |
| 12 | PG(O-16:0/18:4(6Z,9Z,12Z,15Z)) | 746.534 | 11.358 |
| 13 | N-[(4E,8E)-1,3-dihydroxyoctadeca-4,8-dien-2-yl]hexadecanamide | 534.488 | 12.823 |
| 14 | Saponin H | 633.399 | 13.085 |
| 15 | PS(O-20:0/18:3(9Z,12Z,15Z)) | 782.569 | 13.085 |
| 16 | PS (18:3(9Z,12Z,15Z)/19:1(9Z)) | 820.526 | 13.085 |
| 17 | PS(O-18:0/14:0) | 766.539 | 13.257 |
| 18 | N-Palmitoylsphingosine | 536.504 | 13.916 |
| 19 | PG(P-16:0/17:2(9Z,12Z)) | 697.480 | 13.992 |
| 20 | 1-tetradecanoyl-2-(8-[3]-ladderane-octanyl)-sn-glycero-3-phospho-(1'-sn-glycerol) | 697.480 | 13.992 |
| 21 | PS (12:0/15:0) | 648.422 | 14.008 |
| 22 | PS (14:0/12:0) | 669.446 | 14.008 |
| 23 | PS (12:0/17:2(9Z,12Z)) | 672.420 | 14.008 |
| 24 | PE (14:0/18:0) | 690.507 | 14.005 |
| 25 | PE(P-16:0/20:4(5Z,8Z,10E,14Z) (12OH[S])) | 738.507 | 14.005 |
| 26 | PC (14:0/20:2(11Z,14Z)) | 780.551 | 14.008 |
| 27 | PS(P-16:0/22:4(7Z,10Z,13Z,16Z)) | 796.525 | 14.008 |
| 28 | PE (15:0/22:2(13Z,16Z)) | 802.560 | 14.005 |
| 29 | PS (18:3(9Z,12Z,15Z)/19:0) | 822.525 | 822.525 |
| 30 | PS (18:4(6Z,9Z,12Z,15Z)/21:0) | 848.539 | 14.008 |
| 31 | 3-(4-Methyl-3-pentenyl) thiophene | 184.115 | 14.020 |
| 32 | SM(d16:1/17:0) | 687.542 | 14.017 |
| 33 | PE (15:0/18:0) | 706.537 | 14.020 |
| 34 | (+)-Tetrandrine | 623.311 | 3.396 |
| 35 | 20-carboxy Arachidonic Acid | 333.206 | 4.705 |
| 36 | Ile Ile Pro Cys | 445.248 | 5.412 |
| 37 | 9-OxoOTrE | 293.210 | 5.789 |
| 38 | 13S-HpOTrE(gamma) | 293.2108 | 5.876 |
| 39 | 3-Hydroxy-11Z-octadecenoylcarnitine | 442.352 | 5.926 |
| 40 | 3-Hydroxyhexadecanoylcarnitine | 398.326 | 5.988 |
| 41 | Isolinderanolide | 337.273 | 6.146 |
| 42 | MG (0:0/24:6(6Z,9Z,12Z,15Z,18Z,21Z)/0:0) | 448.341 | 6.170 |
| 43 | PG (22:6(4Z,7Z,10Z,13Z,16Z,19Z)/0:0) | 555.272 | 6.292 |
| 44 | (+/-)-14-HDoHE | 343.227 | 6.511 |
| 45 | 15beta-Hydroxydesogestrel | 327.231 | 6.523 |
| 46 | 3-Methylcyclopentadecanone | 239.236 | 6.610 |
| 47 | PGE1 alcohol | 341.268 | 6.610 |
| 48 | Palmitoyl-L-carnitine | 400.341 | 6.610 |
| 49 | 5,7,9,11,13-tetradecapentaenoic acid | 459.247 | 6.634 |
| 50 | (±)12-HETE | 319.227 | 6.633 |
| 51 | LysoPC(16:0) | 540.330 | 6.633 |
| 52 | 3-trans-Caffeoyltormentic acid | 695.382 | 6.633 |
| 53 | PE (18:0/20:3(5Z,8Z,11Z)) | 814.560 | 6.633 |
| 54 | 5(S)-HETE lactone | 605.456 | 6.646 |
| 55 | PE (20:1(11Z)/20:3(5Z,8Z,11Z)) | 840.576 | 6.669 |
| 56 | Ginkgoic acid | 345.243 | 6.753 |
| 57 | Vaccenyl carnitine | 426.357 | 6.803 |
| 58 | (3beta,5alpha,9alpha,22E,24R)-3,5,9-Trihydroxy-23-methylergosta-7,22-dien-6-one | 476.37 | 6.839 |
| 59 | 8(S)-HETrE | 321.243 | 6.900 |
| 60 | PC (17:1(9Z)/20:2(11Z,14Z)) | 842.591 | 6.900 |
| 61 | 8,11,14-Eicosatrienoic acid | 307.264 | 7.179 |
| 62 | 1-Palmitoyl-2-(5-keto-8-oxo-6-octenoyl)-sn-glycero-3-phosphatidylcholine | 648.387 | 7.384 |
| 63 | PI (15:1(9Z)/22:6(4Z,7Z,10Z,13Z,16Z,19Z)) | 884.526 | 7.680 |
| 64 | Polyoxyethylene (600) monoricinoleate | 341.305 | 7.842 |
| 65 | (E)-Suberenol | 538.243 | 7.842 |
| 66 | Azaspiracid | 842.498 | 7.838 |
| 67 | Glycerylphosphorylethanolamine | 196.038 | 8.200 |
| 68 | PS (12:0/20:3(8Z,11Z,14Z)) | 730.465 | 8.348 |
| 69 | PI (15:0/20:5(5Z,8Z,11Z,14Z,17Z)) | 860.527 | 8.436 |
| 70 | PI (13:0/22:6(4Z,7Z,10Z,13Z,16Z,19Z)) | 858.513 | 8.448 |
| 71 | PG (12:0/12:0) | 649.347 | 8.700 |
| 72 | PS (13:0/18:3(9Z,12Z,15Z)) | 698.437 | 8.738 |
| 73 | PS (12:0/17:1(9Z)) | 692.446 | 8.776 |
| 74 | S-(11-hydroxy-9-deoxy-delta12-PGD2)-glutathione | 663.363 | 8.826 |
| 75 | PG (17:1(9Z)/20:4(5Z,8Z,11Z,14Z)) | 783.506 | 9.462 |
| 76 | PC (18:0/22:5(4Z,7Z,10Z,13Z,16Z)) | 874.573 | 9.491 |
| 77 | PI (15:1(9Z)/22:4(7Z,10Z,13Z,16Z)) | 888.557 | 9.503 |
| 78 | 1-(6-[3]-ladderane-hexanoyl)-2-(8-[3]-ladderane-octanyl)-sn-glycerophosphocholine | 826.516 | 9.540 |
| 79 | LysoPC(10:0) | 842.524 | 9.626 |
| 80 | Citroside A | 431.191 | 9.709 |
| 81 | 6'-Hydroxybuspirone | 820.522 | 9.725 |
| 82 | PI (16:0/20:3(5Z,8Z,11Z)) | 859.530 | 9.746 |
| 83 | PI (P-16:0/13:0) | 791.447 | 9.762 |
| 84 | PI (18:1(11Z)/20:4(8Z,11Z,14Z,17Z)) | 885.5489 | 9.887 |
| 85 | PS (18:3(9Z,12Z,15Z)/20:1(11Z)) | 834.525 | 9.914 |
