# Supplementary material for: l-Arginine, as an essential amino acid, is a potential substitute for treating COPD via regulation of ROS/NLRP3/NF-κB signaling pathway
Source: Cell Biosci. 2023 Aug 18;13:152. doi: 10.1186/s13578-023-00994-9 (PMC10436497; doi:10.1186/s13578-023-00994-9)
Supplement: Supplementary file 6 — Additional File 6: Table S2 Quantification of the Top50 different metabolites. [file 13578_2023_994_MOESM6_ESM.docx]

**Additional File 6: Table S2 Quantification of the Top50 different metabolites**

| NO.1 | Identification | Relative contents in Healthy People | Relative contents in COPD People |
| --- | --- | --- | --- |
| 1 | L-Arginine | 281.709±44.241 | 55.361±39.942 |
| 2 | 1-(6-[5]-ladderane-hexanoyl)-2-(8-[3]-ladderane-octanyl)-sn-glycerophosphocholine | 61.440±7.682 | 23.295±4.670 |
| 3 | PE (17:0/12:0) | 397.347±44.241 | 281.709±44.241 |
| 4 | PG (18:1(11Z)/20:4(5Z,8Z,11Z,14Z)) | 1478.574±475.210 | 1203.211±276.264 |
| 5 | PS (18:3(9Z,12Z,15Z)/19:1(9Z)) | 1888.199±349.411 | 1260.876±185.210 |
| 6 | 1-tetradecanoyl-2-(8-[3]-ladderane-octanyl)-sn-glycero-3-phospho-(1'-sn-glycerol) | 800.667±56.968 | 366.055±159.562 |
| 7 | PA (16:0/18:2(9Z,12Z)) | 3378.495±721.524 | 1669.820±222.109 |
| 8 | PE (16:1(9Z)/18:1(11Z)) | 51642.071±4171.469 | 17868.86±6487.898 |
| 9 | PE(O-16:0/20:1(11Z)) | 5768.511±1420.438 | 3886.704±305.618 |
| 10 | PG (15:1(9Z)/18:1(9Z)) | 575.828±149.296 | 264.597±53.977 |
| 11 | PG (18:1(11Z)/18:2(9Z,12Z)) | 4409.471±879.371 | 2619.977±699.406 |
| 12 | PS (18:1(9Z)/18:2(9Z,12Z)) | 978.860±274.810 | 641.251±86.534 |
| 13 | PS (17:1(9Z)/19:1(9Z)) | 897.887±285.500 | 528.621±100.625 |
| 14 | PA (22:4(7Z,10Z,13Z,16Z)/18:0) | 496.254±212.054 | 756.246±73.099 |
| 15 | PS (17:0/17:1(9Z)) | 269.782±120.086 | 388.213±70.167 |
| 16 | PS (20:4(5Z,8Z,11Z,14Z)/18:0) | 1176.155±480.1282 | 1809.642±430.835 |
| 17 | PS (22:4(7Z,10Z,13Z,16Z)/18:0) | 205.125±125.565 | 418.666±69.772 |
| 18 | Dodecanedioic acid | 226.033±32.864 | 260.619±28.876 |
| 19 | Delphinidin 3-sambubioside | 46.481±15.075 | 78.898±10.229 |
| 20 | C16 Sphinganine | 4430.587±625.298 | 5033.887±447.006 |
| 21 | 1,15-Hexadecadien-3-one | 1712.572±154.975 | 2041.433±218.111 |
| 22 | Sphinganine | 173.063±44.039 | 234.999±63.074 |
| 23 | LysoPE(22:6(4Z,7Z,10Z,13Z,16Z,19Z)/0:0) | 6357.308±1168.516 | 4848.978±899.036 |
| 24 | PS (18:3(9Z,12Z,15Z)/0:0) | 67.518±14.32 | 214.103±39.217 |
| 25 | 1-(5Z,8Z,11Z,14Z-eicosatetraenoyl)-sn-glycero-3-phosphate | 263.109±37.216 | 461.72±56.462 |
| 26 | 1-Palmitoyl Lysophosphatidic Acid | 36.261±16.362 | 78.462±19.402 |
| 27 | PC (15:0/22:5(4Z,7Z,10Z,13Z,16Z)) | 186.293±38.617 | 362.369±65.771 |
| 28 | PGE1 alcohol | 1437.791±268.428 | 2518.529±484.416 |
| 29 | Palmitoyl-L-carnitine | 3078.365±528.426 | 4693.083±429.392 |
| 30 | 1-Oleoylglycerophosphoinositol | 1437.791±268.428 | 1437.791±268.428 |
| 31 | Enkephalin L | 3269.317±249.517 | 4970.623±369.52 |
| 32 | Macrocarpal I | 298.461±48.592 | 485.832±76.529 |
| 33 | N-palmitoyl taurine | 579.527±58.665 | 786.582±63.274 |
| 34 | MG (0:0/18:2(9Z,12Z)/0:0) | 157.661±35.782 | 374.932±48.772 |
| 35 | Methantheline | 86.264±14.842 | 139.371±18.382 |
| 36 | PA(P-16:0/20:5(5Z,8Z,11Z,14Z,17Z)) | 1960.804±328.631 | 2549.453±278.873 |
| 37 | Muricoreacin | 1760.803±387.621 | 2984.809±572.316 |
| 38 | Hovenidulcioside B2 | 460.942±87.075 | 875.303±127.321 |
| 39 | Val Ser Glu | 268.482±69.092 | 396.482±78.532 |
| 40 | PA (12:0/18:3(6Z,9Z,12Z)) | 216.392±36.824 | 377.539±54. |
| 41 | Lucidenic acid G | 176.783±54.693 | 376.662±75.093 |
| 42 | Norfloxacin | 268.526±69.662 | 543.084±96.044 |
| 43 | DG (15:0/20:5(5Z,8Z,11Z,14Z,17Z)/0:0) | 216.702±43.615 | 407.731±76.352 |
| 44 | DG (22:4(7Z,10Z,13Z,16Z)/22:6(4Z,7Z,10Z,13Z,16Z,19Z)/0:0) | 186.083±27.482 | 295.428±84.826 |
| 45 | 3-O-Sulfogalactosylceramide (d18:1/18:1(9Z)) | 1364.628±360.605 | 2595.37±426.518 |
| 46 | N-stearoyl taurine | 479.162±60.063 | 764.825±79.742 |
| 47 | PE (14:0/22:5(4Z,7Z,10Z,13Z,16Z)) | 173.281±49.893 | 286.281±76.861 |
| 48 | Oxyphencyclimine | 75.381±13.703 | 147.372±27.629 |
| 49 | Citroside A | 862.631±382.606 | 1530.638±328.631 |
| 50 | Haloperidol | 249.45±47.207 | 428.610±59.274 |
